# Cross-sectoral gerontopsychiatric treatment and rehabilitation in nursing homes (SGB Reha)—study protocol for a stepped-wedge cluster randomized trial

**DOI:** 10.3389/frhs.2026.1895079

**Published:** 2026-10-19

**Authors:** Nora Mierzwa, Björn Gradel, Bernd R. Förstner, Friederike Deeken, Anke Desch, Jennifer M. Schroth, Manuel Du, Oskar Dierbach, Christine Holmberg, Michael Hauptmann, Michael Rapp, Andreas Häusler

**Affiliations:** 1 Social and Preventive Medicine, University of Potsdam, Potsdam, Germany; 2 Institute of Social Medicine and Epidemiology, Brandenburg Medical School Theodor Fontane, Brandenburg an der Havel, Neuruppin, Germany; 3 Institute of Biostatistics and Registry Research, Brandenburg Medical School Theodor Fontane, Neuruppin, Germany; 4 Haus Ruhrgarten, Evangelische Altenhilfe Mülheim an der Ruhr gGmbH, Mülheim an der Ruhr, Germany

**Keywords:** cross-sectoral care, gerontopsychiatry, health services research, implementation science, long-term care, nursing homes, rehabilitation, stepped-wedge cluster randomized trial

## Abstract

**Background:**

Demographic changes in Germany have increased the need for care to 5.7 million people, with around 800,000 living in residential care facilities. Nursing home residents are vulnerable, suffering from multimorbidity and cognitive impairments, with prevalent use of psychotropic medication linked to negative outcomes like sedation and hospitalizations. So far, there is little research on multimodal rehabilitation programs in nursing homes. The SGB Reha study aims to implement a cross-sectoral rehabilitation model to improve activities of daily living (ADLs), cognition, and quality of life for nursing home residents, reducing hospitalizations and treatment costs, and increasing the chance of residents returning home post-care.

**Methods:**

The study is conducted in 12 nursing homes located in North Rhine-Westphalia and Hamburg. It employs a cluster-randomized stepped-wedge design with randomization at the facility level. Data collection occurs at three consecutive measurement points, at 0, 3, and 6 months. The recruitment target includes 720 nursing home residents across four six-month study phases. Nursing home residents aged 65 years and older who had been admitted to the nursing home within the previous 12 months—either as a routine admission or following a hospital stay—and who have a care level of at least 2 are eligible for inclusion. The intervention integrates therapeutic and rehabilitative nursing practices. Structured multidisciplinary case conferences (including neurologists/psychiatrists, and consulting pharmacists) establish objectives, assess progress, adapt personalized care, develop therapeutic strategies, and systematically evaluate medications, especially emphasizing the reduction of psychotropic drugs and polypharmacy prevention. A multidisciplinary team delivers the intervention, supported by study nurses coordinating processes. In addition to resident outcomes, the study evaluates quality of life among relatives and job satisfaction among nursing home staff, allowing assessment of intervention effects across multiple stakeholder groups.

**Discussion:**

The intervention aims to enhance ADLs, cognition, and quality of life for nursing home residents. By combining effectiveness, health-economic, qualitative, and process evaluations, the study adopts a health services perspective that extends beyond resident-level outcomes to include relatives, staff, organizational processes, and healthcare system implications. Results of the trial are intended to contribute to the further development of rehabilitation-oriented and gerontopsychiatric care structures in German nursing homes. From an economic perspective, we anticipate a reduction in treatment costs. This can contribute to a sustainable cross-sectoral care model that integrates therapeutic-rehabilitative approaches into standard care in residential long-term care.

**Trial status:**

Recruitment and data collection are complete; analyses are ongoing (protocol reported after conclusion of data collection).

**Clinical Trial Registration:**

https://www.isrctn.com/ISRCTN11686133, ISRCTN Registry, identifier ISRCTN11686133.

## 1 Introduction

Due to demographic changes, the number of people in need of care continues to increase. In Germany, 5.7 million people need formal or informal care. Around 800,000 individuals currently live in residential care facilities (1). Residents of nursing homes constitute a particularly vulnerable population, characterized by multimorbidity, frailty, cognitive impairments, polypharmacy, and behavioral or psychological symptoms (2). In particular, psychotropic medication use is highly prevalent in German nursing homes and is associated with sedation, falls, hospitalizations, and further cognitive decline (3). At the same time, structured rehabilitative and gerontopsychiatric treatment approaches are insufficiently integrated into routine long-term care.

Especially after extended hospital stays, the risk of requiring permanent full-time residential care increases significantly (4). Furthermore, about 40% of patients who receive short-term care after a hospital stay remain in a long-term care facility (5). To date, evidence on multimodal rehabilitation programs in nursing homes remains limited. Most previous studies were conducted in hospital or post-acute rehabilitation settings. These studies rarely addressed implementation processes, health-economic outcomes, or the integration of gerontopsychiatric expertise into routine nursing home care (6). Evidence from a specialized nursing home rehabilitation program involving 481 residents demonstrated that an intensive cross-sectoral, multidisciplinary rehabilitation concept, including activating nursing care and additional therapeutic support, improved activities of daily living in more than 50% of residents and enabled 68% of participants to return home after discharge, with most remaining in the home environment for at least six months (7). Preliminary analyses of a local best-practice model of therapeutic-rehabilitative care (Haus Ruhrgarten, Evangelische Altenhilfe Mülheim an der Ruhr gGmbH, Mülheim an der Ruhr, Germany) further showed statistically significantly lower values for treatment costs as well as the number and duration of hospital stays compared to similar facilities (8).

The goal of the SGB Reha study is the implementation and multimethod process and outcome evaluation of a cross-sectoral, multidisciplinary rehabilitation concept for older people in nursing homes. SGB Reha is based on a best-practice model of therapeutic-rehabilitative care (8), adapted for implementation in different nursing home facilities. Existing evidence on the effectiveness of such complex therapeutic and rehabilitative interventions for people in nursing homes suggests that they can lead to improved activities of daily living (ADLs), cognitive performance, and quality of life (7). Additionally, improvements at the individual level can improve the quality of life of relatives, increase job satisfaction among nursing staff, and lead to a reduction of inappropriate medications, the number of rehospitalizations, and treatment costs (9, 10).

The SGB Reha intervention specifically addresses nursing home residents following recent hospitalization, a population at particularly high risk for functional decline, loss of independence, cognitive deterioration, hospitalization, and permanent institutionalization. Existing care structures often focus primarily on maintenance of care rather than rehabilitation-oriented and person-centered approaches. SGB Reha therefore aims to strengthen residents' ADLs, mobility, cognition, social participation, and quality of life through a multimodal rehabilitative intervention integrated into routine nursing home care.

We hypothesize that the implementation of the rehabilitative care concept will lead to (i) improved activities of daily living among nursing home residents; (ii) improved job satisfaction among nursing staff as well as improved quality of life among residents and relatives; (iii) reduced hospitalizations and lower treatment-related costs; (iv) a reduction in medication use; and (v) an increased proportion of residents returning to their own homes instead of remaining in long-term residential care.

## 2 Methods and analysis

The presented study protocol is based on the Standard Protocol Items: Recommendations for Interventional Trials (SPIRIT; 11). For additional information to the study, see Additional File S1: SPIRIT Checklist.

### 2.1 Consortium and study group

The consortium leadership and sponsor (AOK Rheinland/Hamburg; German public health insurance) are responsible for the project organization, which primarily includes the temporal and financial coordination of the project. Additionally, it communicates with the Funder (Innovation Fund of the Federal Joint Committee) and engages in regular exchanges with both research institutions and participating nursing homes to promptly address study-related challenges, monitor recruitment progress, and support the implementation of the intervention. The Chair of Social and Preventive Medicine (University of Potsdam) is responsible for the evaluation and data collection. Moreover, it conducts training for the staff of nursing homes participating in the intervention, together with Haus Ruhrgarten (Evangelische Altenhilfe Mülheim an der Ruhr gGmbH). The Institute of Social Medicine and Epidemiology (Brandenburg Medical School) is responsible for the qualitative outcome and process evaluation. The Institute of Biostatistics and Registry Research (Brandenburg Medical School) conducts the analyses of the primary and secondary endpoints as well as the health economic analyses. The German Academy for Gerontopsychiatry and Psychotherapy e. V. (DAGPP) organizes peer consultations in the nursing homes and aids in recruiting neurologists/psychiatrists for case conferences within the framework of the intervention. Finally, a Safety Board is established to monitor the safety of the intervention (see below).

### 2.2 Design

The study follows a cross-sectoral longitudinal design that aims to include 720 residents who have been newly admitted to nursing homes or have been re-admitted following a hospital stay within the last twelve months, 720 relatives, and 180 nursing home staff. The study employs a stepped-wedge design with cluster randomization of twelve German nursing homes in North Rhine-Westphalia and Hamburg, including six urban and six rural facilities. The study will have four periods, each lasting six months (see Figure 1). Data collection for residents and their relatives will take place at three measurement time points within each study period: a baseline assessment at the beginning of the six-month period (T0), a follow-up assessment after three months (T1), and a final assessment after six months (T2). The intervention will be implemented in a stepwise manner, with four nursing homes transitioning per step. During period 1 all nursing homes will remain in the control condition (treatment as usual, TAU). Beginning in period 2, the intervention will be introduced sequentially across groups of nursing homes, such that by period 4 all participating nursing homes will have implemented the intervention. The order in which the nursing homes begin the intervention will be determined using a stratified block randomization, stratified by facility location (urban vs. rural), with two urban and two rural facilities transitioning per step. The stepped-wedge cluster randomized design was selected because it enables the sequential implementation of the intervention under real-world nursing home care conditions while maintaining randomized allocation across facilities. This approach facilitates the staggered introduction of the intervention within routine care processes and accommodates organizational and logistical constraints associated with implementation and staff training. In addition, the design enables the evaluation of intervention effects over time under pragmatic conditions while ensuring that all participating nursing homes ultimately receive the intervention (12).

**Figure 1:**
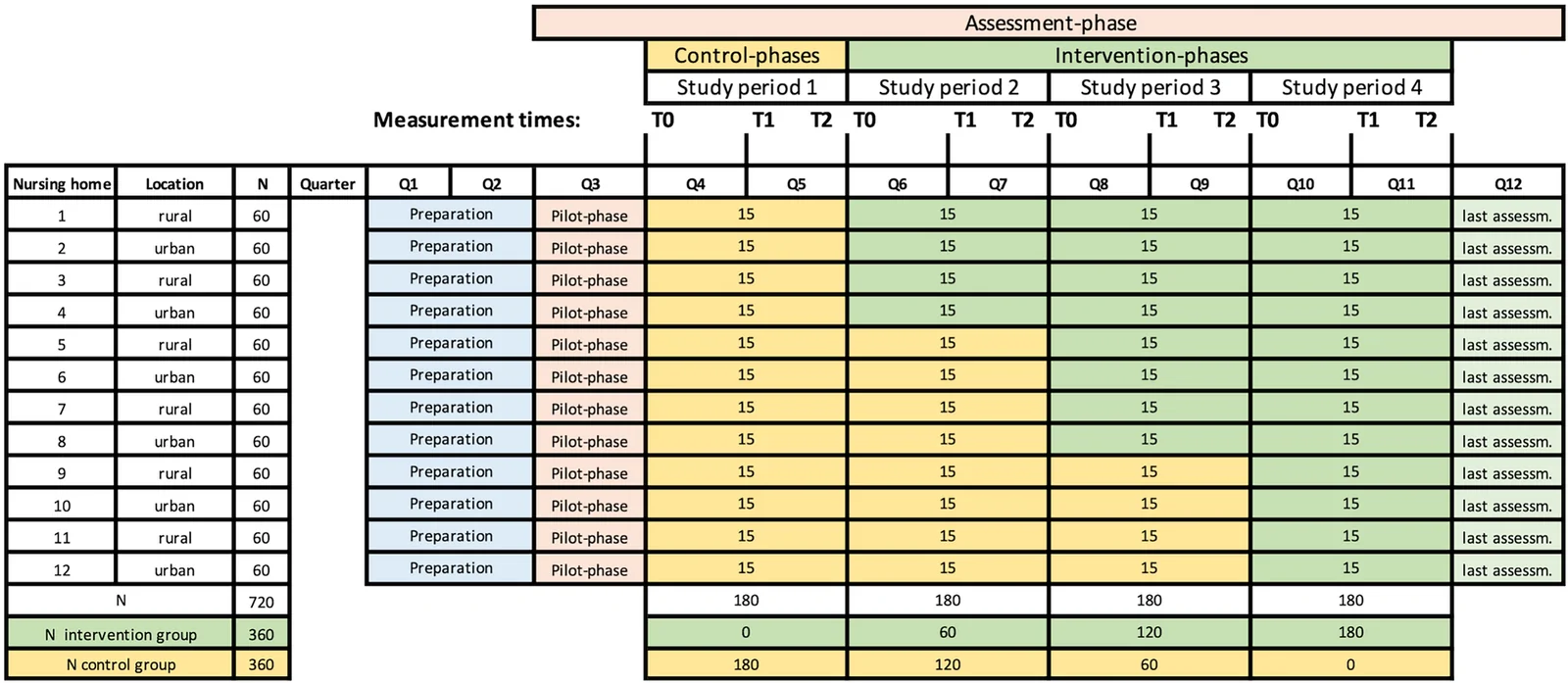
Schematic representation of the planned stepped-wedge cluster-randomized design (control periods in yellow, intervention periods in green). The depicted order is illustrative; the actual crossover order was determined by stratified block randomization, stratified by facility location.

### 2.3 Participants

#### 2.3.1 Inclusion criteria

Eligible nursing home residents must be aged 65 years or older and have been admitted to one of the participating nursing homes within the last twelve months or have been newly admitted to the nursing home following a hospital stay within the last twelve months. Furthermore, insured individuals from the AOK Rheinland/Hamburg statutory health insurance are requested to consent to the disclosure of routine data for health economic analyses. Moreover, we include relatives of participating nursing home residents and staff from the nursing homes. The prerequisite for all study participants is written informed consent (Figure 2).

**Figure 2:**
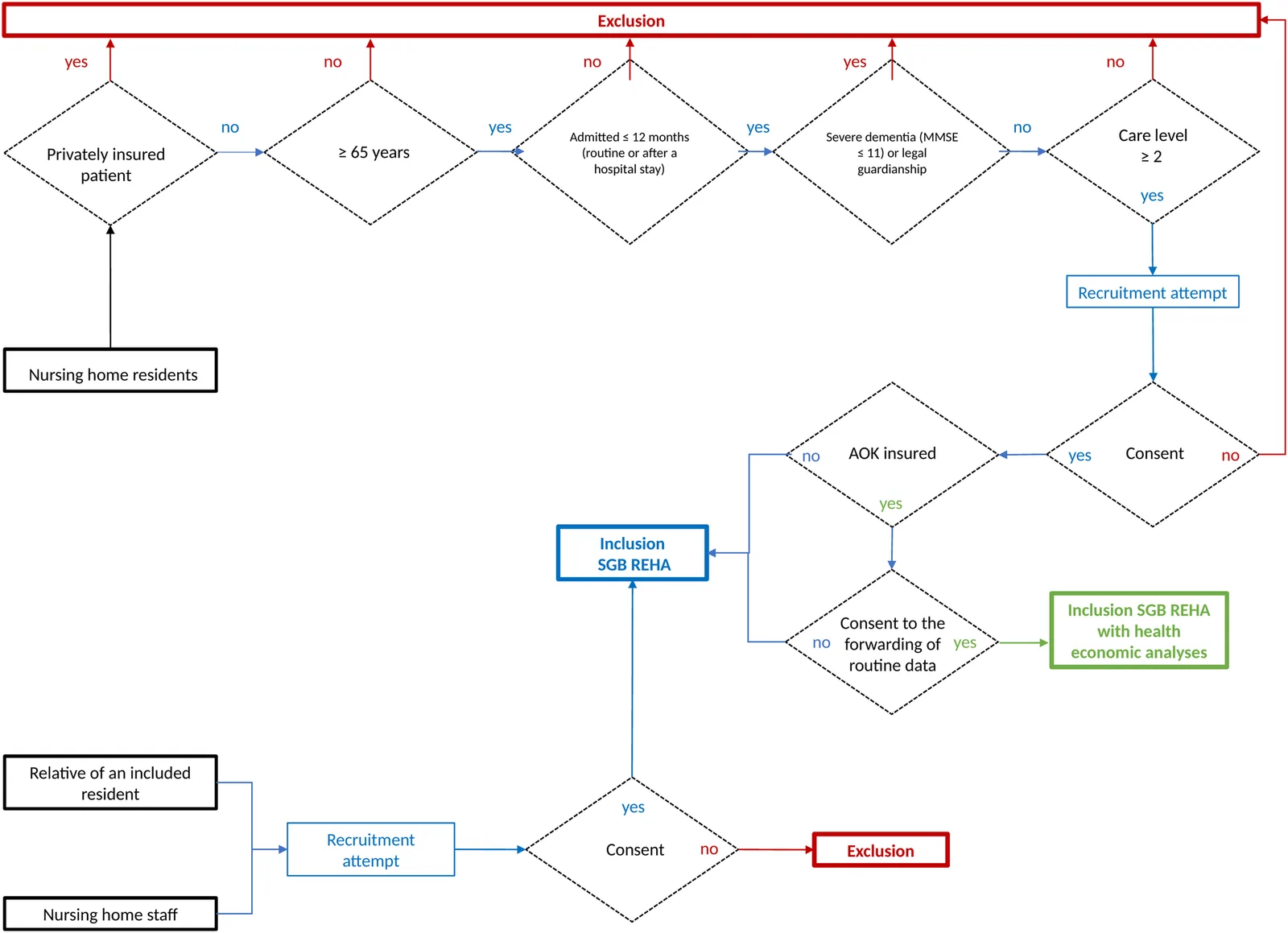
Recruitment algorithm for nursing home residents, relatives, and nursing home staff.

#### 2.3.2 Exclusion criteria

Excluded are nursing home residents who have private health insurance (due to funding restrictions), are unable to give consent due to a severe dementia-related condition (Mini-Mental State Examination (MMSE) ≤ 11 or comparable instrument), are under legal guardianship (§§ 1896 ff. BGB) or have a care level (German “Pflegegrad”) lower than 2. The care level is a statutory classification of care dependency under the German long-term care insurance system, ranging from 1 (minor impairment of independence) to 5 (most severe impairment with special care needs); it is assigned following a standardized assessment by the Medical Service (Medizinischer Dienst). Eligible residents are identified by the facility-based study nurse. Written informed consent is obtained from all participating residents by trained study personnel or study nurses, following oral and written study information and sufficient time to ask questions; no resident is enrolled on the basis of proxy consent. Capacity to consent is assessed prior to enrollment by trained study personnel or study nurses together with the clinical judgment of the nursing team; the MMSE (cut-off ≤11) or a comparable instrument serves only as a screening aid and does not by itself constitute a legal or clinical determination of decision-making capacity. Residents assessed as lacking capacity are not enrolled. Relatives who have at least weekly contact with a participating resident are invited and provide separate written informed consent; in addition, an anonymous relative survey is open to relatives irrespective of whether the resident participates. Nursing home staff working on participating wards are informed and consented separately as an independent cohort. Participation is voluntary for all groups, with the right to withdraw at any time without disadvantage; signed consent forms are stored securely and separately from study data (see also Figure 2). Accordingly, a resident may be included even if their relatives or the nursing staff do not participate.

#### 2.3.3 Expected participant characteristics

As the study includes older adults (≥65 years), a high level of care dependency and substantial heterogeneity in functional status at baseline are expected. Moreover, given the target population, a considerable proportion of residents is anticipated to present with multimorbidity, cognitive impairment, and reduced activities of daily living. Many participants will have been admitted to the nursing home following a recent hospital stay, with lengthy prior hospitalization and underlying diagnoses likely varying across individuals. These baseline characteristics will be assessed and considered as potential confounders in the analyses, as they may influence functional outcomes, health service utilization, and intervention effects.

### 2.4 Intervention

The intervention is based on a theory of change model that was developed in collaboration with stakeholders (8). The intervention is built on a multidisciplinary, rehabilitative care concept with the overarching aim of enhancing residents' everyday functioning, quality of life, mobility and social participation, while simultaneously reducing hospitalizations, care costs and polypharmacy.

The intervention's fundamental approach integrates therapeutic and rehabilitative nursing practices with intensive person-centered care and biographical orientation. A fundamental structural element of the program consists of structured multidisciplinary case conferences held at two-week intervals. These conferences differentiate between “small” and “large” meeting formats, and bring together nursing staff, social care professionals, therapists, neurologists/psychiatrists and consulting pharmacists. They function as the primary medium for the ongoing establishment of objectives, the assessment of progress, the adaptation of personalized care and therapeutic strategies, and the systematic evaluation of medications. A particular emphasis is placed on the reduction of psychotropic medications and the prevention of polypharmacy. Where the prescribing physician participates in the case conference, medication changes—including adjustments of psychotropic drugs by the treating psychiatrist/neurologist—can be initiated directly. Otherwise, the recommendation is forwarded to the treating general practitioner (via the study nurse or nursing staff). In accordance with German law, the consulting pharmacist has no prescribing authority; the final decision and responsibility for any medication change therefore rest with the treating physician, and implementation of a recommendation is not mandated by the study. Because psychotropic medication use and polypharmacy are assessed as outcomes at each measurement time point, the extent to which recommendations translate into actual prescribing changes is captured empirically. In addition, nursing staff receive training in rehabilitative care and the handling of polypharmacy prior to each facility's intervention phase.

Each resident is provided with therapeutic services that are drawn from a minimum of two of the three defined clusters: physiotherapy/motor therapy, occupational therapy, or music/art therapy. Care, therapy and social participation activities are closely integrated into daily routines to ensure continuity and ecological validity of the rehabilitative approach. The concept is characterized by its emphasis on individualization. Care and therapeutic plans are tailored on the basis of comprehensive medical and biographical assessments, considering each resident's functional, psychosocial and emotional needs, as well as their personal preferences and participation goals. Any adjustments made are systematically documented and reviewed in subsequent case conferences.

The intervention is delivered by a multidisciplinary team comprising facility nursing and social-care staff trained in rehabilitative care, in-house and external therapists, neurologists/psychiatrists, and a contracted consulting pharmacist as an integral member; general practitioners remain the residents' treating physicians. It is funded by the Innovation Fund of the Federal Joint Committee (G-BA); the consortium leader (AOK Rheinland/Hamburg) disburses an intervention lump sum to the participating facilities, which primarily finances additional intervention-related personnel (chiefly additional nursing and therapy capacity) as well as the contracted therapeutic and medical-psychiatric services. Study nurses coordinate the processes at each facility and support adherence to trial requirements, including assessor blinding; the German Academy for Gerontopsychiatry and Psychotherapy (DAGPP) supports the recruitment of neurologists/psychiatrists for the case conferences. To ensure that the additional intervention activities are delivered as intended, each facility receives a two-day training before its intervention phase, and peer consultation is provided throughout to support implementation fidelity, motivation, and problem-solving. Delivery of the intervention is recorded in the case-conference documentation (maintained by the study nurse) and in the nursing and therapy documentation (maintained by nursing staff and therapists), which serve as indicators of intervention dose and fidelity (see Section 2.5).

The control condition is defined as Treatment-as-Usual (TAU), reflecting standard nursing home practice without modification. This encompasses routine nursing and medical care as provided by each facility, along with usual social care offerings. Facilities assigned to the control condition do not implement a structured multidisciplinary rehabilitative program, nor do they engage in systematic medication optimization processes or structured integration of therapeutic services into daily nursing care as defined by the intervention protocol. The standard care provided at each nursing home remains unchanged until it transitions to the intervention phase, in accordance with the stepped-wedge design.

### 2.5 Adherence to intervention

Each participating nursing home receives training for the intervention implementation before their respective intervention period starts. A support structure via e-mail and telephone is provided. Adherence to the intervention is monitored through both quantitative documentation and qualitative process evaluation. Quantitatively, the intervention documentation records the frequency and the participating professional groups of the multidisciplinary case conferences, the additional therapeutic services delivered, and the peer-consultation sessions, providing indicators of intervention dose and fidelity. Qualitatively, staff members' subjective experiences of implementation barriers and intervention compliance are collected through semi-structured interviews, and intervention activities are examined through participant observation (conducted by the Brandenburg Medical School Theodor Fontane). Further methodological detail on the process evaluation, including the Organizational Readiness for Implementing Change (ORIC) questionnaire and analysis using the Consolidated Framework for Implementation Research (CFIR), is provided in Section 2.14.3 (Process evaluation).

### 2.6 Adverse events

Given the predominantly non-pharmacological and low-risk nature of the intervention, it is not anticipated that there will be any serious adverse events directly attributable to the study procedures. Nevertheless, for the residents in the intervention group minor risks are associated due to increased therapy and possible medication changes. A possible reduction of medication is only considered following a detailed medical review involving the general practitioner, psychiatrist/neurologist, pharmacist, and communication with residents. Any specific medication adjustments can only be implemented with the approval of the treating physician/general practitioner, as the responsibility for treatment decisions remains with them. The safety and well-being of all participants will be continuously monitored throughout the trial.

An independent external Safety Board has been established to evaluate participant safety at regular intervals. The Board convenes every six months to review the safety of the intervention and to assess whether the trial should continue as planned, be modified, or terminated. The Safety Board consists of two national experts in gerontopsychiatry nominated by the German Academy for Gerontopsychiatry and Psychotherapy e. V. (DAGPP): Prof. Andreas Fellgiebel, MD, and Jochen Tenter, MD. Both members are independent of the sponsor and the trial investigators and have declared no competing interests.

Any adverse events occurring during the study period will be documented and reported to the Safety Board in a timely manner. Study-related adverse events are first assessed by the consortium leadership and then forwarded to the independent Safety Board, which decides on any further steps, including modification or termination of the trial. The Board's safety decisions are authoritative and are not subject to being overridden by the sponsor or the coordinating body; the consortium leader and project management are responsible only for the operational implementation of a Board decision.

### 2.7 Withdrawal

Participants may withdraw from the study at any time without providing reasons and may request the deletion of their data at any point during the study. Withdrawal will not result in any disadvantage to the participant.

Specific discontinuation criteria apply to each participant group. For *residents*, participation will be terminated in the event of death, revocation of consent, discharge to home care, or other reasons deemed appropriate by the investigative team. For *relatives*, discontinuation will occur upon the death of the resident with whom they are associated, revocation of their own consent, or other reasons. For *employees*, participation will be terminated upon revocation of consent or for other applicable reasons. Data collected prior to withdrawal will be retained and included in the analysis unless the participant explicitly requests deletion.

### 2.8 Outcome assessment

Most assessments are conducted by trained assessors who are blinded to each facility's allocation status (see Section 2.12, Randomization). To protect this blinding, information that could reveal a facility's intervention status, such as current medication lists and the medical history (anamnesis), is collected by the study nurses rather than by the blinded assessors. Reliability and plausibility checks will be performed by the University of Potsdam to ensure consistency of measurement across sites and time points. An overview of all instruments and their respective assessment times is provided in Table 1.

**Table 1:** Assessment instruments.

Test	One study period (6 months)		
	T0	T1 (after 3 months)	T2 (after 6 months)
Assessment of the residents			
Sociodemographic assessment	X		
Smoking habits	X		
Alcohol consumption	X		
Recent falls	X	X	X
Level of care	X		
Dominant hand	X		
Initial Anamnesis	X		
Follow-up Anamnesis		X	X
SINGER	X	X	X
Barthel-Index short	X	X	X
TFI	X	X	X
MoCA	X	X	X
20-Cents Test	X	X	X
SF-12	X	X	X
WHOQOL-OLD	X	X	X
Handgrip Strength	X	X	X
MNA-SF	X	X	X
Medication list—Psychotropic drugs	X	X	X
Medication list—other drugs	X	X	X
Assessment of the relatives			
Sociodemographic assessment	X		
WHOQOL-BREF	X	X	X
Assessment of the employees			
Sociodemographic assessment	X (only at the start of the Study)		
COPSOQ	X (at the start of each period)		

SINGER, Scores of Independence for Neurologic and Geriatric Rehabilitation; TFI, Tilburg Frailty Indicator; MoCA, Montreal Cognitive Assessment; SF-12, Short Form 12 Health Survey; MNA-SF, Short Form of the Mini Nutritional Assessment; COPSOQ, Copenhagen Psychosocial Questionnaire. Barthel-Index short, Barthel Index short form (three items); WHOQOL-BREF, World Health Organization Quality of Life – BREF (abbreviated version); WHOQOL-OLD, World Health Organization Quality of Life – Older Adults Module.

#### 2.8.1 Primary outcome

The primary outcome will be ADL skills of the nursing home residents, measured by the Scores of Independence for Neurologic and Geriatric Rehabilitation (SINGER) (13). The SINGER is based on the International Classification of Functioning, Disability and Health (ICF) and assesses 20 aspects of independence in daily activities of living. The development study reported excellent internal consistency (Cronbach's *α* = 0.97) and interrater reliability (ICC = 0.996) and demonstrated strong convergent validity with other commonly used rating instruments, including the Barthel Index (BI; *r* = 0.907 with the physical activities factor of the SINGER) and the Functional Independence Measure (FIM; *r* = 0.980) (13). Compared with the BI and FIM, the SINGER showed considerably reduced ceiling effects in an outpatient neurorehabilitation setting, indicating good discriminative capacity (14).

#### 2.8.2 Secondary outcomes

##### 2.8.2.1 Residents

The secondary outcomes for nursing home residents are frailty, cognitive abilities, independence in activities of daily living, health-related quality of life, fine motor skills, age-related quality of life, physical fitness, nutritional state, number of hospital stays, psychotropic medication in defined daily dosage (DDD), polypharmacy, and proportion of residents discharged to their own homes. These will be measured with the Tilburg Frailty Indicator (TFI) for frailty (15), the Montreal Cognitive Assessment (MoCA) for cognitive impairment (16), the short form of the Barthel Index (three items) for independence in activities of daily living (17), the 12-Item Short-Form Health Survey (SF-12) for health-related quality of life (18), the 20-Cents-Test for fine motor skills (19), the WHOQOL-OLD for age-related quality of life (20), hand grip strength for physical fitness and the Short Form of the Mini Nutritional Assessment (MNA-SF) for nutritional state (21). The number of hospital stays, psychotropic medication (in DDD), polypharmacy, and discharge to home are derived from clinical and routine (claims) documentation.

At baseline (T0), the assessment is supplemented by a comprehensive sociodemographic and clinical evaluation. Sociodemographic information collected includes age, gender, marital status, number of children, immigration background, native language, educational level, occupation, living arrangements, nicotine and alcohol consumption, history of falls, level of care dependency, and dominant hand. Comorbidities are quantified using the expanded version of the Charlson Comorbidity Index (CCI; 22), alongside documentation of hospital stays in the last 12 months and a neurological evaluation. Medication intake is recorded separately for psychotropic drugs and other medications to evaluate polypharmacy. At T1 (three months after baseline) and T2 (six months after baseline), the assessment battery is largely repeated. Semi-structured interviews are conducted in each intervention phase to qualitatively evaluate residents' subjective experience of the intervention. The full sociodemographic evaluation is not readministered in its entirety; however recent falls are reassessed at each follow-up time point, and the initial anamnesis is replaced by follow-up assessments documenting hospital stays in the preceding three months.

##### 2.8.2.2 Relatives

The secondary outcome for relatives is quality of life, assessed at all three time points (T0, T1, and T2) using the WHOQOL-BREF (23). Semi-structured interviews are conducted in each intervention phase to qualitatively evaluate relatives' subjective experience of the intervention. A sociodemographic assessment is additionally conducted at baseline.

##### 2.8.2.3 Employees

The secondary outcome for employees is job satisfaction, evaluated using the Copenhagen Psychosocial Questionnaire (COPSOQ) at the start of each study period (24). Semi-structured interviews are conducted prior to and in each intervention phase to qualitatively evaluate employees' subjective experience of the intervention and implementation factors. A sociodemographic assessment is conducted at the beginning of the study.

##### 2.8.2.4 Health-economic analyses (cost analysis)

For planned health-economic analyses, treatment costs of each participating nursing home will be assessed alongside health insurance costs for each resident. The latter will be evaluated in the subgroup of residents insured with AOK Rheinland/Hamburg.

### 2.9 Timelines

The first enrollment began in April 2023 (study period 1), and the final enrollment began in October 2024 (study period 4). The last assessments were completed in April/May 2025. At the time of writing, data collection is complete and the effectiveness and health-economic analyses are ongoing (see also Figure 1).

Unlike the stepped-wedge assessment schedule applied to residents and relatives, nursing home staff were assessed at five measurement time points (T0–T4). The first four assessments (T0–T3) were conducted (starting April 2023) at the stepped-wedge baseline measurement point (T0) of each respective study period, while the final assessment (T4) took place at the end of the last intervention phase (in April 2025).

### 2.10 Sample size

Based on preliminary qualitative studies, we expect 15–40 new admissions per nursing home over a six-month period, of which on average 15 residents per cluster can be included per period. Across 12 clusters and four periods, this corresponds to a recruitment target of 720 residents (12 × 4 × 15). After deducting an anticipated dropout of 30% of the 15 residents per cluster and period, approximately 10 residents per cluster per period—about 480 residents in total (12 × 4 × 10)—are expected to be available for analysis. The sample-size and power calculation was performed for the primary outcome (SINGER) using PASS (25) for the stepped-wedge cluster-randomized design with 12 clusters and four periods, assuming a standardized intervention effect of 0.4 standard deviations, a two-sided significance level of 5%, and an intracluster correlation coefficient of 0.01. Under these assumptions, the design provides approximately 80% power to detect the intervention effect, based on the Wald test for the treatment coefficient of the linear mixed-effects model. The calculation was based on the number of residents expected to be available for analysis (approximately 480), targeting the absolute change in the SINGER score from the start to the end of a study period; the within-phase longitudinal structure (three measurement points) was not separately modelled and no additional intra-subject correlation was specified, which is not expected to materially affect the power.

### 2.11 Recruitment

The study nurse, based in each nursing home (employed 6 months before to 3 months after the assessment periods; a total of 33 months regardless of randomization status), functions as a central link within the facility, thereby enabling the targeted recruitment of residents. It is imperative to explore alternative strategies for achieving the target sample size if needed. One such strategy could be to increase the number of cases in parallel facilities. If this proves to be ineffective, a subsequent strategy could be to recruit additional facilities. The underlying factors contributing to any encountered recruitment challenges will be systematically investigated at an early stage through the qualitative investigations into implementation barriers and solutions will be developed.

### 2.12 Randomization

Randomization was conducted at the cluster level (*k* = 12 nursing homes) as a stratified block randomization, stratified by facility location (rural/small-town vs. large-city/urban), using Castor EDC, with the allocation sequence generated by the scientific staff of the University of Potsdam. At each of the three crossover steps, four facilities (two urban and two rural) were randomly selected to begin the intervention. During implementation, one small-town/rural facility withdrew and was replaced by a large-city facility; the intended urban/rural balance could therefore not be fully maintained (see deviations, Discussion). Because implementing the intervention requires participating facilities to create additional staff positions and to arrange training schedules in advance, each nursing home and the training team are informed of the facility's allocated study phase (control or intervention) six months before the start of the respective phase. Consequently, nursing home staff, residents, and relatives are necessarily aware of their facility's allocation status, and blinding at this level is not feasible in this pragmatic, cluster-level design. The complete randomization sequence is held only by the coordinating scientific staff at the University of Potsdam and Brandenburg Medical School.

To maintain blinding of outcome assessment, outcome assessors (raters) are not members of the nursing home staff and are not involved in intervention delivery or training. The true aim of the trial is concealed from the raters, who are informed that the purpose is a psychometric validation of the assessment instruments; the on-site study nurses conceal the current intervention status of each facility; and staff, residents, and relatives are instructed not to disclose the interventional nature of the trial to the raters. Nevertheless, because the intervention operates at the facility level and entails visible changes to care processes, complete assessor blinding cannot be guaranteed; this residual risk is acknowledged in the Limitations.

### 2.13 Data management

#### 2.13.1 Data entry and coding

Data entry and coding are conducted utilizing secuTrial®, a certified data capture system compliant with Good Clinical Practice (GCP) standards and FDA 21 CFR Part 11. The system automates the generation of pseudonyms (Study-ID) for new study participants upon enrollment, ensuring consistency and minimizing errors. Secure transmission between the tablets used for assessments and the secuTrial® server is maintained through 128-bit SSL encryption, protecting data integrity during transfer. The study nurses in respective facilities maintain a specialized study identification list, containing pseudonyms, full names, birth dates, addresses, phone numbers, and enrollment dates. These lists enable person identification, which is essential for subsequent assessments. Measures to reduce errors include strict adherence to coding protocols and regular updates by study nurses.

#### 2.13.2 Data security

Data security is paramount, with all study data and participant information secured by multifactor authentication processes for authorized study personnel only. Access to the study server is restricted, ensuring that personnel can only view and manage data specific to their assigned roles and permissions. The transmission of sensitive information, including insurance numbers and health data, is facilitated by encrypted channels utilizing Cryptshare. Furthermore, the separation of contact data from study data is meticulously maintained through the implementation of a storage protocol that utilizes separate, secure cabinets, thereby reducing the risk of unauthorized access or mishandling.

#### 2.13.3 Data storage and time frame

The data collected during the course of the study are stored exclusively on servers located in Germany, in compliance with federal and regional data protection laws (BlnDSG, BbgDSG) and the General Data Protection Regulation (GDPR). The data undergo a mandatory storage period of ten years following the conclusion and publication of the study results, as per the standards of Good Scientific Practice. Subsequent to the conclusion of the study, the secuTrial® license will reach its expiration date, thereby ensuring the removal of all data from its servers following the export process. Two copies of the anonymized study data archive are secured, with password protection, within the University of Potsdam for the stipulated duration. These data management procedures ensure the confidentiality, integrity, and availability of study data while facilitating effective analysis and reporting. For complete information regarding data management practices, please refer to the study Data Management Plan, which is not included in this protocol.

### 2.14 Data analysis

#### 2.14.1 Main analysis method for statistical comparison

The statistical analysis will utilize repeated-measures linear mixed-effects multilevel models, incorporating nursing home clusters as random effects to account for intra-cluster correlation and periods as fixed effects to adjust for temporal trends. The statistical models will provide effect estimates for the intervention across primary and secondary endpoints. These endpoints include basic and instrumental activities of daily living, frailty, cognitive function, hand strength, fine motor skills, health-related quality of life, nutritional state, job satisfaction of the employees and relatives' quality of life. These estimates will be reported with 95% confidence intervals to quantify the precision of effect sizes and allow for interpretation of statistical significance. The models are estimated by restricted maximum likelihood (REML); analyses are conducted at a significance level of 0.05, with a Kenward-Roger adjustment for the degrees of freedom and further adjustments for multiple testing where necessary. Sensitivity analyses will include interaction terms to examine potential heterogeneity in intervention effects across different participant groups. For both the primary outcome (improvement in the SINGER score) and all secondary endpoints, adjusted and non-adjusted analyses will be performed according to the intention-to-treat principle. Primary analyses will focus on adjusted results to present a robust outcome assessment, using age and sex as prespecified covariates. On an exploratory basis, additional covariates such as care level, cognitive impairment, or depression will be considered and included as needed. No imputation is performed; missing outcome data are accommodated by the likelihood-based linear mixed-effects model, which uses all available measurements under a missing-at-random assumption. Data on age and sex are expected to be complete, and analyses involving further potential covariates are performed on complete cases only.

#### 2.14.2 Health economic analyses

The health-economic evaluation is a cost analysis comparing healthcare costs with intervention costs from the perspective of the statutory health insurance (payer perspective). Health economic analyses will be performed for the sub cohort of individuals insured with AOK Rheinland/Hamburg, using routine claims data and adopting the perspective of the statutory health insurance (payer perspective). Sector-specific and total healthcare expenditures will be assessed at the individual level, comprising the following categories: outpatient care, inpatient care, prescription drugs, and other healthcare services (including allied health services, medical aids and devices, and patient transportation). Costs incurred outside the statutory health insurance (e.g., societal or informal care costs) are not captured. The effect of the intervention on healthcare expenditures will be analyzed using mixed-effects models that account for the skewed distribution of cost data (e.g., generalized linear models with a Gamma distribution). The estimated difference in average healthcare expenditures per person between the intervention and control conditions will then be compared with the average per-person intervention costs (e.g., additional therapy, case conferences, and training) to assess whether the intervention offsets its own costs. No formal cost-effectiveness or cost-utility analysis (e.g., cost per QALY) is planned, as preference-based utility measures were not collected. Sensitivity analyses will be conducted to evaluate the robustness of the results.

#### 2.14.3 Process evaluation

The process evaluation identifies barriers and facilitators for implementation on the contextual, organizational, and individual level. Data is gathered in four one-week observation periods per facility prior to and during each intervention phase. During these, semi-structured interviews and the German version of the Organizational Readiness for Implementing Change (ORIC) questionnaire (26), provided by the ImpleMentAll Consortium (new reference), are conducted with staff members, and observations of work routines and intervention measures are made. Barriers and facilitators are analyzed using the updated Consolidated Framework for Implementation Research (CFIR; 28).

#### 2.14.4 Qualitative outcome evaluation

For the qualitative outcome evaluation, three one-week observation periods per facility are conducted at the end of each intervention phase. The intervention's subjective impact on study participants will be captured and analyzed through semi-structured interviews with participants, relatives and staff, and participant observations in the facilities. Qualitative data is analyzed deductively and inductively in MAXQDA to evaluate anticipated as well as unexpected intended and unintended outcomes and revise the intervention's theory of change model (8).

The results of the process and qualitative outcome analysis will be analyzed for congruence with quantitative results. This will facilitate a holistic evaluation of intervention success and long-term applicability.

## 3 Discussion

This study has several important strengths. The SGB Reha trial evaluates a complex cross-sectoral, multidisciplinary gerontopsychiatric rehabilitation intervention in nursing homes using a multicenter stepped-wedge cluster randomized design. Despite increasing numbers of older adults requiring institutional long-term care, structured rehabilitative and gerontopsychiatric treatment concepts remain insufficiently integrated into routine nursing home care in Germany (2).

Another major strength of the study is the development and evaluation of a multidisciplinary intervention combining rehabilitative nursing, therapeutic services, structured medication review, psychosocial activation, and multidisciplinary case conferences. Particular emphasis is placed on reducing psychotropic medication use and polypharmacy while improving individualized and rehabilitation-oriented care processes. Existing evidence suggests that multidisciplinary rehabilitative interventions can improve functional outcomes, independence, and quality of life in older adults living in long-term care settings (6, 7, 10, 29, 30). Furthermore, the intervention specifically addresses nursing home residents following recent hospitalization, a population at increased risk for functional decline, loss of independence, cognitive deterioration, and permanent institutionalization (4, 5).

An additional strength of the study is the use of a stepped-wedge cluster randomized design. This design enables evaluation of intervention effects both within and between participating nursing homes while accounting for temporal and organizational influences. Compared to non-randomized implementation studies, stepped-wedge cluster randomized trials provide more robust evidence for complex healthcare interventions (12). Importantly, the design ensures that all participating nursing homes receive the intervention by the end of the study, thereby improving feasibility and ethical acceptability.

The study also integrates quantitative effectiveness analyses, health-economic evaluation, and qualitative process and outcome evaluation. This mixed-methods approach enables not only the assessment of intervention effectiveness, but also the investigation of implementation barriers, facilitators, and contextual influences within routine long-term care settings. The use of the Organizational Readiness for Implementing Change (ORIC) framework and the Consolidated Framework for Implementation Research (CFIR) further supports a comprehensive understanding of implementation processes and contextual determinants of intervention success (26, 27, 28). In addition, perspectives of residents, relatives, and nursing home staff are included, allowing a multidimensional evaluation of intervention effects.

This manuscript reports the study protocol as originally planned and prospectively registered (ISRCTN11686133; registered 27 March 2023, prior to first enrollment) and approved by the ethics committee (E-02-20220930; 19 December 2022). At the time of writing, recruitment and data collection are complete and the analyses are ongoing. To ensure transparency, we distinguish: (i) the originally planned protocol, described here; (ii) approved amendments made during trial conduct; (iii) operational deviations, notably the withdrawal and replacement of one facility that affected the planned urban/rural balance; and (iv) any analyses added after completion of recruitment, which will be explicitly labelled as *post hoc*. The amendments to the ethics-approved protocol and the operational deviations are summarised in Table 2; the trial registration (ISRCTN11686133) was not changed after initial registration. Any further deviations arising during implementation will be reported transparently in the forthcoming outcome publications, ensuring a clear separation between the *a priori* study plan and its real-world implementation.

**Table 2:** Amendments to the ethics-approved protocol and operational deviations.

#	Type	Date	Description	Status
1	Ethics amendment	Apr–May 2023	Participant information and informed-consent forms (residents) revised to comply with the Declaration of Helsinki (explicit right to withdraw at any time without notice).	Acknowledged by the ethics committee
2	Ethics amendment	25 Apr 2023	Document updates at the start of the first study period, including the health-economic analysis and AOK routine-data provisions.	Acknowledged by the ethics committee
3	Ethics amendment	18 Sep 2023	Relative inclusion criteria expanded: relatives of any resident (irrespective of the resident's study participation) and more than one relative per resident may be included, to enlarge the recruitment base and to capture possible spill-over effects; new relative information/consent documents.	Acknowledged by the ethics committee
4	Ethics amendment	Mar–Apr 2024	Addition of the anonymous relative survey and documentation for the main study.	Acknowledged by the ethics committee
D1	Operational deviation	2023	One small-town/rural facility withdrew and was replaced by a large-city facility; the planned urban/rural balance could therefore not be fully maintained.	To be detailed in the outcome publication
—	Trial registration	—	ISRCTN11686133: no changes to the registered record after initial registration (27 March 2023).	—

Several limitations should nevertheless be considered. First, the intervention itself is highly complex and consists of multiple interacting components implemented within routine nursing home care. While this pragmatic approach enhances real-world applicability, it also makes it difficult to determine which specific intervention components are responsible for observed effects. Furthermore, participating facilities are intentionally granted a degree of flexibility in implementing the intervention to accommodate local organizational conditions. As a result, intervention fidelity may vary across nursing homes and may contribute to heterogeneity in outcomes. Taken together with the eligibility restrictions described below (exclusion of residents with severe dementia and of those with a care level below 2), this complexity also limits the external validity and generalizability of the findings to the wider nursing home population. In addition, with only 12 clusters, inference from the multilevel models may be sensitive to model specification and to the degrees-of-freedom approximation; the models are therefore estimated by REML with a Kenward-Roger correction, and sensitivity analyses are reported.

Due to the nature of the intervention, blinding of nursing home staff and participants is not feasible. Although assessor blinding procedures are implemented, complete prevention of unblinding cannot be guaranteed. In addition, implementation fidelity may differ between facilities due to variations in staffing levels, organizational culture, leadership structures, and available resources.

Another limitation concerns participant recruitment. Recruitment was conducted after cluster randomization and relied on study nurses within participating nursing homes to identify eligible residents. Although clearly defined inclusion criteria restricted participation to residents newly admitted within the previous twelve months, a certain degree of selection bias cannot be excluded.

A further limitation is the exclusion of residents with severe dementia-related inability to provide informed consent, including those under legal guardianship (§§1896 ff. BGB). This decision was made on ethical grounds, in line with the responsible ethics committee, and also reflects the rehabilitative, person-centered nature of the intervention, which relies on residents' active participation and the articulation of individual goals. Consequently, the findings may not be fully generalizable to the most vulnerable nursing home populations, who often have the highest burden of gerontopsychiatric symptoms and functional impairment.

The study will also investigate whether improvements in quality of life and functional independence can be achieved without increasing healthcare expenditures. Given the close relationship between functional decline, hospitalization, polypharmacy, and gerontopsychiatric symptoms in nursing home residents (2, 3), improving rehabilitative and multidisciplinary care may represent an important strategy to improve outcomes and reduce long-term healthcare costs.

Finally, the results of the study are intended to contribute to the further development of rehabilitation-oriented and gerontopsychiatric care structures in German nursing homes. If effective, the intervention may provide an evidence base for sustainable cross-sectoral models of care and inform future recommendations and healthcare policy regarding therapeutic-rehabilitative long-term care. Furthermore, the findings may support the implementation of rehabilitation-oriented care concepts in routine nursing home practice and strengthen the integration of gerontopsychiatric expertise into long-term care settings. Ultimately, SGB Reha may contribute to the evidence base for person-centered and rehabilitation-oriented models of care and support future efforts to improve functional independence, quality of life, and social participation among nursing home residents.

## 4 Ethics and dissemination

The study was approved by the Ethics Committee of the Brandenburg Medical School Theodor Fontane, Neuruppin, Germany (reference number: E-02-20220930, approved 19 December 2022). The trial is conducted in accordance with the Declaration of Helsinki, Good Clinical Practice principles, and applicable German data protection regulations, including the General Data Protection Regulation (GDPR). The trial was prospectively registered in the ISRCTN registry (ISRCTN11686133). The exclusion of residents unable to provide personal informed consent, including those under legal guardianship (§§1896 ff. BGB), and the non-use of proxy consent were part of the study design approved by the ethics committee (E-02-20220930).

Written informed consent is obtained from all participants prior to study inclusion. Residents unable to provide informed consent (e.g., due to severe dementia-related cognitive impairment or legal guardianship) are excluded from participation. Participation is voluntary and may be withdrawn at any time without disadvantage.

The intervention is based on established therapeutic-rehabilitative and gerontopsychiatric care principles implemented within routine nursing home care structures. No separate participant (trial) insurance (Probandenversicherung) was arranged, consistent with the approved ethics application, given the non-interventional, low-risk nature of the study; staff are covered through their employing institutions, and existing institutional and statutory insurance coverage applies.

The results of the study will be disseminated through peer-reviewed scientific publications, conference presentations, workshops, and reports to participating nursing homes and healthcare stakeholders. Authorship eligibility follows International Committee of Medical Journal Editors (ICMJE) recommendations. Professional medical writers will not be involved in manuscript preparation.

In addition to scientific dissemination, the project aims to facilitate implementation of rehabilitation-oriented and gerontopsychiatric care concepts into routine nursing home practice. Findings from the process and outcome evaluation will therefore be used to develop practical implementation strategies, training materials, and dissemination formats for nursing homes and healthcare professionals.

If successful, the SGB Reha intervention may contribute to strengthening cross-sectoral rehabilitative care structures for older adults in need of long-term care and support the further development of therapeutic-rehabilitative care approaches within German nursing homes.

## Data Availability

Upon reasonable request, an anonymized data set may be made available, subject to approval by the responsible data protection office and applicable consent. The code is available from the authors upon reasonable request.
