# Supplementary material for: Cross-sectoral gerontopsychiatric treatment and rehabilitation in nursing homes (SGB Reha)—study protocol for a stepped-wedge cluster randomized trial
Source: Front Health Serv. 2026 Oct 19;6:1895079. doi: 10.3389/frhs.2026.1895079 (PMC13724501; doi:10.3389/frhs.2026.1895079)
Supplement: Supplementary file 1 [file Datasheet1.pdf]

Section/Item	Item	Description	
**Administrative Information**			
Title and structured summary	1a	Title stating the trial design, population, and interventions, with identification as a protocol	Cross-sectoral gerontopsychiatric treatment and rehabilitation in nursing homes (SGB Reha) - study protocol for a stepped-wedge cluster randomized trial
	1b	Structured summary of trial design and methods, including items from the World Health Organization Trial Registration Data Set	**Primary Registry and Trial Identifying Number:**<br>ISRCTN Registry – ISRCTN11686133<br>**Secondary Identifying Numbers:**<br>Innovation Fund project number: 01NVF21108<br>**Source(s) of Monetary or Material Support:**<br>This study is funded by the Innovation Fund of the Federal Joint Committee (Gemeinsamer Bundesausschuss, G-BA), Germany.<br>**Primary Sponsor:**<br>AOK Rheinland/Hamburg – Die Gesundheitskasse, Düsseldorf, Germany<br>**Contact for public Queries:**<br>Ms Isabella Heller<br>Press Spokesperson<br>AOK Rheinland/Hamburg – Die Gesundheitskasse<br>Kasernenstraße 61<br>40213 Düsseldorf<br>Germany<br>Phone: +49 (0) 211 8791 28474<br>Email: presse@rh.aok.de

Section/Item	Item	Description	
			**Contact for Scientific Queries:**<br>Andreas Häusler<br>Social and Preventive Medicine<br>University of Potsdam<br>Am Mühlenberg 9<br>14476 Potsdam<br>Germany<br>Phone +49 331 977 4185<br>Fax +49 331 977 4078<br>Andreas.haeusler@uni-potsdam.de<br>Orcid-ID: 0000-0003-1963-8373<br><br>**Public Title:**<br>Sektorenübergreifende gerontopsychiatrische Behandlung und Rehabilitation in Pflegeheimen: SGB-REHA [Interdisciplinary geriatric psychiatric treatment and rehabilitation in care homes: SGB-REHA]<br><br>**Scientific Title:**<br>Cross-sectoral gerontopsychiatric treatment and rehabilitation in nursing homes (SGB Reha) - study protocol for a stepped-wedge cluster randomized trial<br><br>**Countries of Recruitment:**<br>Germany.<br><br>**Health Condition(s) or Problem(s) Studied:**<br>Elderly, multimorbid long-term residents of nursing homes, their independently living relatives, and nursing home staff.

Section/Item	Item	Description	
			**Study Type:**<br>Interventional stepped-wedge cluster-randomized superiority trial.<br><br>**Date of First Enrollment (planned):**<br>1 April 2023 (prospectively registered on 27 March 2023)<br><br>**Sample Size:**<br>720 participants (planned), with approximately 15 residents per cluster per 6-month period<br><br>**Study Objectives:**<br>The implementation of the rehabilitation concept aims to:<br>1. Improve residents' activities of daily living skills<br>2. Increase job satisfaction among nursing staff and quality of life among residents and relatives<br>3. Reduce hospitalizations and treatment costs<br>4. Reduce pharmaceutical use<br>5. Increase the number of residents returning to their own homes<br><br>**Key Inclusion Criteria:**<br>Resident of one of the 12 participating nursing homes<br>● Age ≥ 65 years<br>● Newly admitted or readmitted after a hospital stay within the previous twelve months

Section/Item	Item	Description	
			• Care level ≥ 2<br>• Written informed consent<br><br>**Key Exclusion Criteria:**<br>• Severe dementia (MMSE ≤ 11 or comparable assessment)<br>• Lack of capacity to provide informed consent without legal representative<br>• Private health insurance<br><br>**Intervention:**<br>The intervention consists of a complex multimodal cross-sectoral rehabilitative program including:<br>• Interdisciplinary extended team meetings (additional involvement of a pharmacist and/or neurologist/psychiatrist)<br>• Staff training (2-day sessions) in therapeutic-rehabilitative care<br>• Extended therapy offers (physiotherapy, occupational therapy, music/art therapy)<br>• Therapeutic guidance for nurses<br>• Person-centered multi-professional rehabilitation model<br>• Strengthening of social/day-structuring activities<br>• Team supervision<br>• Enhanced communication with relatives<br>The stepped-wedge design includes three sequential crossover phases at 6-month intervals across 12 nursing homes.

Section/Item	Item	Description	
			**Comparator:**<br>Usual care during control periods of the stepped-wedge design.<br><br>**Primary Outcome:**<br>Change in activities of daily living measured by the SINGER scale at baseline (T0), 12 weeks (T1), and 24 weeks (T2)<br><br>**Key Secondary Outcomes:**<br>Cognition (Montreal Cognitive Assessment), frailty (Tilburg Frailty Indicator), health-related quality of life (SF-12), hospitalizations, psychotropic medication use (defined daily dosage), polypharmacy, discharge to home, staff job satisfaction (COPSOQ), quality of life of relatives, and health-economic outcomes<br><br>**Ethics Review:**<br>Approved 19 December 2022. Ethics Committee of the Brandenburg Medical School Theodor Fontane. Reference: E-02-20220930<br><br>**Individual Participant Data (IPD) Sharing Statement:**<br>There are no plans to share individual participant data. Access to study data is restricted in accordance with data protection regulations and contractual agreements with the sponsor.
Protocol version	2	Version date and identifier	V 2.0, 05. September 2025.<br>This version reflects updates to the evaluation concept, recruitment procedures, and inclusion criteria. Any future protocol amendments will be documented with version number, date, and description of changes and will be communicated to the ethics committee and trial registry.

Section/Item	Item	Description	
Roles and responsibilities	3a	Names, affiliations, and roles of protocol contributors	• **Prof. Dr. Dr. Michael Rapp**, Department of Social and Preventive Medicine, University of Potsdam: study conception, study design, supervision of quantitative evaluation, and overall scientific coordination.<br><br>• **Prof. Dr. Christine Holmberg**, Institute of Social Medicine and Epidemiology, Brandenburg Medical School (MHB), Neuruppin: qualitative evaluation, process evaluation based on the Theory of Change framework, and implementation research.<br><br>• **Prof. Dr. Michael Hauptmann**, Institute of Biometry and Registry Research, Brandenburg Medical School (MHB), Neuruppin: statistical design, sample size calculation, development of the statistical analysis plan, and independent biometrical evaluation.<br><br>• **Charlotte Boutmans**, AOK Rheinland/Hamburg – Die Gesundheitskasse, Düsseldorf: consortium management, project coordination, contractual implementation, and provision of routine health-economic data.<br><br>• **Oskar Dierbach**, Evangelische Altenhilfe Mülheim an der Ruhr (Haus Ruhrgarten): implementation support and transfer of the therapeutic-rehabilitative nursing model to participating nursing homes.<br><br>• **Dr. Thomas Kunczik**, German Academy for Gerontopsychiatry and Psychotherapy (DAGPP): medical-psychiatric expertise and structured training components within the intervention.<br><br>Roles within Protocol:<br>MR, CH, MH developed the study design. AH, BF, and AD developed the evaluation design, the minimum intervention set and the trainingtraining concept (in cooperation with O. Dierbach). BG and NM wrote the first draft of the manuscript. Authors BG and NM contributed equally to the first draft of the manuscript. All authors contributed to and have approved the final manuscript.

Section/Item	Item	Description	
	3b	Name and contact information for the trial sponsor	**Trial Sponsor:**<br>AOK Rheinland/Hamburg – Die Gesundheitskasse<br><br>**Sponsor Address:**<br>Kasernenstraße 61<br>40213 Düsseldorf<br>Germany<br><br>**Sponsor Contact Person:**<br>Charlotte Boutmans<br>AOK Rheinland/Hamburg – Die Gesundheitskasse<br><br>Telephone: +49 211 8791 28042<br>Email: charlotte.boutmans@rh.aok.de<br><br>**Regulatory Agency Identifying Number:**<br>Not applicable. This is a non-pharmacological health services research trial and does not require authorization by a medicines regulatory authority.
	3c	Role of trial sponsor and funders in design, conduct, analysis, and reporting of trial; including any authority over these activities	The Federal Joint Committee (G-BA) holds the innovation fund which is funding the study, but has no role in the design of this study and will not have any role during its execution, analyses, interpretation of the data, or decision to submit results.
	3d	Composition, roles, and responsibilities of the coordinating site, steering committee, endpoint adjudication committee, data management team, and other individuals or groups overseeing the trial, if applicable	The consortium leader, **Charlotte Boutmans (AOK Rheinland/Hamburg – Die Gesundheitskasse)**, has the following roles:<br><br>• The management of the overall project and its subprojects, monitoring progress according to the planned schedule. The consortium leader may refuse the acceptance of work packages or reports and request revisions where necessary.<br>• Representation of the project in communication with the Innovation Fund of the Federal Joint Committee (G-BA), and fulfilment of all reporting obligations.<br>• Coordination of communication between the Steering Committee and the project partners, particularly regarding joint publications and milestone reporting.

Section/Item	Item	Description	
			• Oversight of contractual implementation and allocation of funding to consortium partners.<br><br>The Steering Committee consists of:<br><br>1. **Charlotte Boutmans** (AOK Rheinland/Hamburg, consortium leader)<br>2. **Prof. Dr. Dr. Michael Rapp** (University of Potsdam)<br>3. **Prof. Dr. Christine Holmberg** (Brandenburg Medical School, MHB)<br><br>4. **Prof. Dr. Michael Hauptmann** (Brandenburg Medical School, MHB)<br><br>Led by the consortium leader, the Steering Committee meets at least twice per year.<br><br>The Steering Committee has the following roles:<br><br>• Writing the minutes of meetings of the project committee and project management and making them available to all partners.<br>• Coordination, collection, storage, and distribution of all documents and information relevant to the project.<br>• Cooperation with the project committee and implementation of its decisions.<br><br>Further tasks include:<br><br>• Managing the scientific and operational development of the project.<br>• Assisting the consortium leader in preparing reports for the sponsor.<br>• Coordinating joint publications and requests for data analysis and submitting them to the project committee for approval.<br>• Addressing operational and scientific issues relevant to trial conduct.<br><br>The Project Committee consists of one representative per consortium partner:<br><br>1. **Charlotte Boutmans** (AOK Rheinland/Hamburg)

Section/Item	Item	Description	
			2. **Prof. Dr. Dr. Michael Rapp** (University of Potsdam)<br>3. **Prof. Dr. Christine Holmberg** (MHB)<br>4. **Prof. Dr. Michael Hauptmann** (MHB)<br>5. **Dr. Thomas Kunczik** (German Academy for Gerontopsychiatry and Psychotherapy, DAGPP)<br><br>6. **Oskar Dierbach** (Evangelische Altenhilfe Mülheim an der Ruhr, Haus Ruhrgarten)<br><br>Led by the consortium leader, the Project Committee meets at least biannually.<br><br>Its roles are:<br><br>• Deciding on the admission or exclusion of project partners (subject to sponsor regulations).<br>• Deciding on substantial protocol amendments.<br>• Verifying appropriate transfer and use of funding between consortium partners.<br>• Assisting in preparation and approval of reports to the sponsor.<br>• Coordinating joint publications and data analysis requests.<br>• Addressing circumstances relevant to the scientific and operational development of the project.<br><br>The statistical analysis is conducted independently by the **Institute of Biometry and Registry Research (MHB)** under the leadership of **Prof. Dr. Michael Hauptmann**.<br><br>The statistical team is responsible for:<br><br>• Development of the statistical analysis plan<br>• Independent analysis of primary and secondary outcomes<br>• Sensitivity analyses and subgroup analyses<br>• Preparation of statistical reports

Section/Item	Item	Description	
			The sponsor has no role in statistical analysis, interpretation of data, or decision to publish results.<br><br>An independent monitoring board will be established to oversee study progress and quality assurance.<br><br>Its responsibilities include:<br><br>• Periodic review of recruitment, protocol adherence, and milestone achievement<br>• Review of safety-relevant parameters (e.g., falls, morbidity, mortality)<br>• Advisory recommendations to the Steering Committee<br><br>The monitoring board acts independently of both the sponsor and the academic investigators.
**Open science**			
Trial registration	4	Name of trial registry, identifying number (with URL), and date of registration. If not yet registered, name of intended registry	**Name of registry:**<br>ISRCTN Registry<br><br>**Trial registry identifying number:**<br>ISRCTN11686133<br><br>**URL to registry record:**<br>https://www.isrctn.com/ISRCTN11686133<br><br>**Date of registration:**<br>27 March 2023 (prospectively registered prior to participant enrollment; first enrollment on 1 April 2023).
Protocol and statistical analysis plan	5	Where the trial protocol and statistical analysis plan can be accessed	The full study protocol will be published in a peer-reviewed scientific journal.

Section/Item	Item	Description	
			In addition, key protocol elements are publicly accessible via the ISRCTN registry (ISRCTN11686133).<br><br>The full statistical analysis plan (SAP) will be finalized prior to database lock and will be made available upon reasonable request from the corresponding author.
Data sharing	6	Where and how the individual de-identified participant data (including data dictionary), statistical code, and any other materials will be accessible	Individual de-identified participant data, including the data dictionary, will not be made publicly available.<br><br>Due to German data protection regulations (including the General Data Protection Regulation, GDPR) and contractual agreements with the trial sponsor, participant-level data cannot be shared with external parties.<br><br>Statistical code used for the primary and secondary analyses may be made available upon reasonable request to the corresponding author, provided that such sharing complies with institutional and sponsor regulations.<br><br>Materials associated with the intervention (e.g., training materials and implementation manuals) may be shared upon reasonable request for academic, non-commercial purposes.<br><br>No additional consent for public data sharing will be obtained from participants.
Funding and conflicts of interest	7a	Sources of funding and other support (e.g., supply of drugs)	**Name of funder:**<br>Innovation Fund of the Federal Joint Committee (Gemeinsamer Bundesausschuss, G-BA), Germany.<br><br>**Type of funding:**<br>Direct monetary support.<br><br>The Innovation Fund provides full financial support for the implementation and evaluation of the study, including personnel costs, data collection, qualitative and

Section/Item	Item	Description	
			quantitative analyses, and health-economic evaluation.<br><br>**Name of sponsor:**<br>AOK Rheinland/Hamburg – Die Gesundheitskasse, Germany.<br><br>**Type of support:**<br>Indirect support.<br><br>The sponsor provides routine health insurance data for health-economic analyses and supports contractual implementation of the intervention within participating nursing homes.<br><br>**Other support:** No pharmaceutical products, medical devices, or other commercial products are supplied free of charge.<br>No professional medical writers are involved.<br>No additional external funding sources contribute to the study.
	7b	Financial and other conflicts of interest for principal investigators and steering committee members	The principal investigators and members of the Steering Committee declare no financial conflicts of interest related to the intervention under study.<br><br>None of the investigators:<br>● Hold stocks or shares related to the intervention<br>● Receive honoraria, consultancy payments, or advisory board fees related to the intervention<br>● Hold patents or pending patents related to the intervention<br><br>The study is funded by the Innovation Fund of the Federal Joint Committee (G-BA). The trial sponsor, AOK Rheinland/Hamburg – Die Gesundheitskasse, is a statutory health insurance provider and participates as consortium leader.<br><br>As the sponsor provides routine health insurance data for health-economic analyses,

Section/Item	Item	Description	
			a potential institutional interest in cost-related outcomes cannot be fully excluded. However, the following measures are implemented to reduce potential influence:<br>● The statistical analysis is conducted independently by the Institute of Biostatistics and Registry Research (MHB).<br>● The statistical analysis plan is finalized prior to database lock.<br>● The sponsor has no role in statistical analysis, interpretation of results, or the decision to publish.<br>● Scientific reporting is led by the academic investigators.<br>No additional non-financial conflicts of interest (e.g., advocacy roles or personal affiliations) have been declared.
Dissemination policy	8	Plans to communicate trial results to participants, healthcare professionals, the public, and other relevant groups (e.g., reporting in trial registry, plain language summary, publication)	The results of the trial will be disseminated to participants, healthcare professionals, policymakers, and the broader scientific community through multiple channels.<br>**Communication to Participants and Nursinghomes**: Participating nursing homes will receive a structured summary of the study results. A plain language summary of the main findings will be prepared and made available to residents and their relatives upon request. Study results will be presented to participating facilities in feedback workshops.<br>**Communication to healthcare professionals and stakeholders**: Findings will be presented at national and international scientific conferences in geriatrics, gerontopsychiatry, health services research, and implementation science. Results will also be shared with relevant professional associations and health insurance stakeholders.<br>**Scientific Publication**: The study protocol and primary results will be submitted to peer-reviewed scientific journals. Open access publication will be pursued whenever possible. Secondary analyses (e.g., qualitative and health-economic results) will be published separately.

Section/Item	Item	Description	
			**Trial Registry Reporting**: The ISRCTN registry entry (ISRCTN11686133) will be updated with summary results after completion of the trial.<br><br>**Public Communication**: The sponsor (AOK Rheinland/Hamburg) may issue press releases in coordination with the academic investigators. Public communication will reflect the scientific results and will not precede peer-reviewed publication.<br><br>The sponsor and funder will have no authority to prevent publication of the trial results.
**Introduction**			
Background and rationale	9a	Scientific background and rationale, including summary of relevant studies (published and unpublished) examining benefits and harms for each intervention	Due to demographic changes, the number of people in need of care continues to increase: In Germany, 5.7 million people need formal or informal care. Around 800,000 of these are cared for in residential care facilities (1). Residents of nursing homes constitute a particularly vulnerable population, characterized by multimorbidity, frailty, cognitive impairments, polypharmacy, and behavioral or psychological symptoms (2). Specifically, the use of psychotropic medications is widespread in German nursing homes and is associated with sedation, falls, hospitalizations, and further cognitive decline (3). At the same time, structured rehabilitative and gerontopsychiatric treatment approaches are insufficiently integrated into routine long-term care.<br><br>Especially after extended hospital stays, the risk of requiring permanent full-time residential care increases significantly (4). Furthermore, about 40% of patients who receive short-term care after a hospital stay remain in a long-term care facility (5). So far, there is little research on multimodal rehabilitation programs in nursing homes: Most of the prior research was carried out in hospital or post-acute rehabilitation environments, lacking a systematic focus on implementation processes, health-economic impacts, or the incorporation of gerontopsychiatric expertise into standard care practices within nursing homes (6). Evidence from a specialized nursing home rehabilitation program involving 481 residents demonstrated that an intensive cross-sectoral, multiprofessional rehabilitation concept, including activating

Section/Item	Item	Description	
			nursing care and additional therapeutic support, improved activities of daily living in more than 50% of residents and enabled 68% of participants to return home after discharge, with most remaining in the home environment for at least six months (7). Preliminary analyses of a local best-practice model of therapeutic-rehabilitative care (Haus Ruhrgarten, Mülheim an der Ruhr, Germany) further showed statistically significantly lower values for treatment costs as well as the number and duration of hospital stays compared to similar facilities.<br><br>The goal of the SGB Reha study is the implementation and multimethod process and outcome evaluation of a cross-sectoral, multiprofessional rehabilitation concept for older people in nursing homes. SGB Reha is based on a best-practice model of therapeutic-rehabilitative care (8), adapted for implementation in different nursing home facilities. Existing evidence on the effectiveness of such complex-therapeutic, rehabilitative interventions for people in nursing homes suggests that they can lead to improved activities of daily living (ADLs), cognitive performance, and quality of life (7). Additionally, ameliorations on individual levels can improve the quality of life of relatives, increase job satisfaction among nursing staff, and lead to a reduction of inappropriate medications, the number of rehospitalizations, and treatment costs (9, 10).<br><br>The SGB Reha intervention specifically addresses nursing home residents following recent hospitalization, a population at particularly high risk for functional decline, loss of independence, cognitive deterioration, hospitalization, and permanent institutionalization. Existing care structures often focus primarily on maintenance of care rather than rehabilitation-oriented and person-centered approaches. SGB Reha therefore aims to strengthen residents' ADLs, mobility, cognition, social participation, and quality of life through a multimodal rehabilitative intervention integrated into routine nursing home care.<br><br>We hypothesize that the implementation of the rehabilitative care concept will lead to (i) improved activities of daily living among nursing home residents; (ii) improved job satisfaction among nursing staff as well as improved quality of life among residents and relatives; (iii) reduced hospitalizations and lower treatment-related

Section/Item	Item	Description	
			costs; (iv) a reduction in medication use; and (v) an increased proportion of residents returning to their own homes instead of remaining in long-term residential care.
	9b	Explanation for choice of comparator	The comparator condition in the present study is usual care provided in participating nursing homes during the control periods of the stepped-wedge design.<br><br>Usual care in German nursing homes typically includes standard nursing care, routine medical treatment by general practitioners and specialists, and access to therapeutic services according to existing regulations and local availability. However, structured multidisciplinary rehabilitative concepts integrating gerontopsychiatric, therapeutic, nursing, and psychosocial components are not routinely implemented across facilities.<br><br>The use of usual care as comparator was chosen for several reasons. First, no established standard for cross-sectoral gerontopsychiatric rehabilitation in nursing homes currently exists in Germany. Second, comparison against routine care allows evaluation of the additional effectiveness and implementation feasibility of the SGB Reha intervention under real-world conditions. Third, the stepped-wedge cluster-randomized design ensures that all participating nursing homes eventually receive the intervention, which was considered ethically appropriate given the anticipated benefits of the rehabilitative care concept.<br><br>The comparator therefore reflects current routine practice and enables assessment of whether the intervention provides clinically meaningful improvements beyond existing care structures.
Objectives	10	Specific objectives related to benefits and harms	The primary objective of the SGB Reha trial is to evaluate the effectiveness of a cross-sectoral, multidisciplinary gerontopsychiatric rehabilitation concept in improving activities of daily living among nursing home residents.

Section/Item	Item	Description	
			The secondary objectives are to evaluate whether the intervention:<br><br>1. Improves cognitive functioning, mobility, frailty status, nutritional status, and health-related quality of life among nursing home residents;<br>2. Reduces hospitalizations, psychotropic medication use, polypharmacy, and treatment-related healthcare costs;<br>3. Increases the proportion of residents returning to their own homes instead of remaining in permanent residential care;<br>4. Improves quality of life among residents' relatives and job satisfaction among nursing home staff;<br>5. Demonstrates cost-effectiveness compared to usual care;<br>6. Can be successfully implemented in routine nursing home care and integrated into existing cross-sectoral care structures.<br><br>The study further aims to investigate implementation processes, contextual factors, and mechanisms of action through embedded qualitative process and outcome evaluation.<br><br>As the intervention is non-pharmacological and based on established rehabilitative and psychosocial approaches, no major intervention-related harms are anticipated. Potential adverse events, including falls, hospitalization, morbidity, and mortality, will nevertheless be monitored throughout the study.
**Methods: Patient and public involvement, trial design**			
Patient and public involvement	11	Details of, or plans for, patient or public involvement in the design, conduct, and reporting of the trial	Patients and members of the public were involved indirectly in the development of the intervention through the best-practice therapeutic-rehabilitative care model on which the SGB Reha project is based. Experiences and practical expertise from nursing home residents, relatives, nursing staff, therapists, and care providers informed the development of the multidisciplinary rehabilitation concept and its implementation procedures.<br><br>In addition, participating nursing homes and practice partners contributed to the

Section/Item	Item	Description	
			planning and operational adaptation of the intervention to routine long-term care settings. The intervention was designed to address the specific needs of nursing home residents with functional and gerontopsychiatric impairments and to strengthen communication between residents, relatives, and care staff.<br><br>Residents, relatives, and nursing home staff are actively involved in the qualitative process and outcome evaluation through semi-structured interviews and participant observations. Their perspectives will contribute to the evaluation of implementation processes, acceptability, perceived intervention effects, and contextual factors influencing trial outcomes.<br><br>The results of the study will be communicated to participating nursing homes, residents, relatives, and healthcare professionals through structured summaries, feedback workshops, scientific publications, and conference presentations. A plain language summary of the study findings will be made available upon request.
Trial design	12	Description of trial design including type of trial (e.g., parallel group, crossover), allocation ratio, and framework (e.g., superiority, equivalence, non-inferiority, exploratory)	The SGB Reha study is a multicenter stepped-wedge cluster-randomized interventional trial with embedded qualitative process and outcome evaluation.<br>The study aims to include 720 nursing home residents, 720 relatives, and 180 nursing home staff members across twelve participating nursing homes in Rhineland-Palatinate and Hamburg, Germany, including six urban and six rural facilities.<br>The study follows a stepped-wedge cluster-randomized superiority design with four consecutive study periods of six months each. Following an initial 6-month control phase in all participating nursing homes, clusters sequentially transition from the control condition (treatment as usual, TAU) to the intervention condition in three implementation waves at 6-month intervals. In each step, four randomly selected nursing homes cross over to the intervention until all facilities have implemented the intervention by Period 4.<br>The order of crossover is determined using stratified block randomization according to urban and rural facility location. The allocation ratio across the three implementation waves is 1:1:1.

Section/Item	Item	Description	
			Outcome assessments are conducted at baseline (T0), after three months (T1), and after six months (T2) within each study period.<br>The stepped-wedge cluster-randomized design was selected because the intervention is implemented at facility level and requires sequential staff training and organizational implementation under routine care conditions. Furthermore, the design allows pragmatic evaluation of intervention effects while ensuring that all participating nursing homes eventually receive the intervention.
**Methods: Participants, interventions, and outcomes**			
Trial setting	13	Settings (e.g., community, hospital) and locations (e.g., countries, sites) where the trial will be conducted	The study takes place in 12 nursing homes in the federal states of North Rhine-Westphalia and Hamburg, Germany. Six of these nursing homes are located in urban areas whereas the other six are located in rural areas.
Eligibility criteria	14a	Eligibility criteria for participants	**Residents** of the nursing homes are eligible to participate if they are aged over 65 years, have been newly admitted or readmitted to the nursing home after a hospitalisation in the last 12 months, have been assigned at least care level 2 according to § 15 SGB XI and have provided informed consent. Patients are excluded if they have severe dementia (MMST ≤ 11 or similar instrument/assessment of the nursing team), lack the capacity to consent or do not wish to participate in the study, have legal guardianship according to §§1896 ff. BGB or have private health insurance (funder requirement).<br><br>**Relatives** are eligible for participation if they are a relative of a participating resident, defined as someone who has at least weekly contact with them, and if they provide informed consent. For the anonymous survey of the relatives, all relatives of the nursing home residents are eligible and the residents did not have to participate in the study. Relatives were excluded if they were relatives of an excluded resident or did not provide informed consent.<br><br>**Employees** of the nursing homes are eligible for participation if they work on wards that are part of the intervention and provide informed consent. Exclusion criteria

Section/Item	Item	Description	
			include not working on wards that are part of the intervention or not providing informed consent.
	14b	If applicable, eligibility criteria for sites and for individuals who will deliver the interventions (e.g., surgeons, physiotherapists)	Several nursing homes in North Rhine-Westphalia and Hamburg were contacted if they wanted to participate in the study. If they wanted to participate, they had to submit a letter of intent.
Intervention and comparator	15a	Intervention and comparator with sufficient details to allow replication including how, when, and by whom they will be administered. If relevant, where additional materials describing the intervention and comparator (e.g., intervention manual) can be accessed	**Intervention Group:**<br>The intervention is a multidisciplinary, rehabilitative care concept for nursing home residents with gerontopsychiatric and geriatric conditions. It aims to enhance everyday functioning, quality of life, mobility, participation and also to reduce hospitalizations, care costs and polypharmacy.<br><br>Components:<br>- Therapeutic-rehabilitative nursing based on intensive person-centered care and biographical orientation<br>- Structured multiprofessional case conferences every two weeks ("small" and "large" meetings) involving nursing, social care, therapists, external neurologists/psychiatrists and a consulting pharmacist<br>- Systematic medication review with a specific emphasis on reducing psychotropic medication and preventing polypharmacy<br>- Therapeutic services from at least two of the following clusters: physiotherapy/mototherapy, occupational therapy, and music/art therapy<br>- Continuous goal setting, evaluation and adaptation of care and therapeutic plans<br>- Close integration of care, therapy, and social participation activities into daily routines<br>- Peer consultation to support implementation, motivation and problem solving

Section/Item	Item	Description	
			How the intervention is administered and by whom:<br>- Nursing staff and social care professionals trained in rehabilitative care<br>- External and in-house therapists<br>- Neurologists/psychiatrists and a contractual pharmacist<br>- Supported by Study Nurses coordinating processes and ensuring trial requirements (including blinding of assessors)<br>- all other nursing home staff in contact with the residents<br><br>Tailoring / individualization<br>- Comprehensive medical and biographical assessment<br>- Individual functional, psychosocial and emotional needs<br>- Resident preferences and participation goals<br>- Adjustments are documented and reviewed continuously in subsequent case conferences<br><br>**Control Group:**<br>The comparator is Treatment-as-Usual (TAU). This consists of:<br>- Standard nursing and medical care according to routine facility practice<br>- Usual social care offerings<br>- No structured multiprofessional rehabilitative program, no systematic medication optimization processes as defined in the intervention and no structured therapeutic integration into daily nursing care<br>- Usual care continues unchanged until each facility crosses over to the intervention phase within the stepped-wedge design
	15b	Criteria for discontinuing or modifying allocated intervention/comparator for a trial participant (e.g., drug dose change in response to harms, participant request, or improving/worsening disease)	Cancellation criteria for residents:<br>- Death<br>- Revocation of consent to participate in the study<br>- Discharge to home care<br>- other reasons<br><br>Cancellation criteria for relatives:

Section/Item	Item	Description	
			- Death of relatives resident<br>- Revocation of consent to participate in the study<br>- other reasons<br><br>Cancellation criteria for employees:<br>- Revocation of consent to participate in the study<br>- Job change<br>- other reasons
	15c	Strategies to improve adherence to intervention/comparator protocols, if applicable, and any procedures for monitoring adherence (e.g., drug tablet return, sessions attended)	Employees of each participating nursing home receive training for the intervention implementation before their first respective intervention period starts. A support structure via email and telephone is provided. Case conferences, additional therapeutic expenditures and peer consultation are documented.<br>Adherence to the intervention and implementation difficulties are also monitored through a qualitative assessment by the Medizinische Hochschule Brandenburg.
	15d	Concomitant care that is permitted or prohibited during the trial	None.
Outcomes	16	Primary and secondary outcomes, including the specific measurement variable (e.g., systolic blood pressure), analysis metric (e.g., change from baseline, final value, time to event), method of aggregation (e.g., median, proportion), and time point for each outcome	**Primary outcome:**<br>ADL skills of the residents, measured by Scores of Independence for Neurologic and Geriatric Rehabilitation (SINGER) for baseline as well as after 3 and 6 months.<br><br>**Secondary outcomes:**<br>Measures of Frailty (Tilburg Frailty Indicator (TFI)), Cognition (Montreal Cognitive Assessment (MoCA)), health-related quality of life (Short Form 12 Health Survey (SF-12)), fine-motor skills (20-Cents-Test), general quality of life (WHOQOL-OLD), physical fitness (hand-grip strength), nutritional state (Mini Nutritional Assessment (MNA)), number of hospital stays, psychopharmaceutical medication in defined daily dosage (DDD), polypharmacy and proportion of residents discharged to their own homes are secondary outcomes for *residents*. All secondary outcomes for residents are measured at baseline as well as after 3 and 6 months.

Section/Item	Item	Description	
			Measures of job satisfaction (Copenhagen Psychosocial Questionnaire (COPSOQ) are secondary outcomes for the *employees* and general quality of life (WHOQOL-BREF) for the *relatives*. The secondary outcomes for the employees are measured at baseline and then every 6 months at the start of each new study period while the secondary outcomes for the relatives are measured at baseline at the start of a period as well as after 3 and 6 months.<br><br>**For baseline assessment:**<br>Sociodemographic assessment, smoking behaviour, alcohol consumption, falls in the last three months, level of care, dominant hand and anamnesis (incl. Charlson Comorbidity Index (CCI)).<br><br>**For planned health-economic analyses:**<br>Treatment costs of each respective nursing home, health insurance cost of each resident (in the subgroup of AOK Rheinland/Hamburg patients)<br><br>**For process analyses/evaluation of results:**<br>Process-related outcomes are specified as secondary outcomes and address intervention fidelity, implementation quality, contextual conditions, and mechanisms of impact in accordance with the Theory of Change framework. These outcomes are not part of a core outcome set.<br><br>Process outcomes include organizational and contextual variables (infrastructure, resident characteristics, working culture), delivery and realization of intervention components, and perceived barriers and facilitators, assessed via structured observations and semi-structured interviews.<br><br>Outcomes are analyzed longitudinally at the facility level (pre-implementation and during implementation), summarized using qualitative thematic analyses and cross-facility comparisons. Data are collected prior to implementation, throughout the intervention period, and approximately three months after implementation, and

Section/Item	Item	Description	
			are triangulated with quantitative effectiveness outcomes to contextualize trial results.
Harms	17	How harms are defined and will be assessed (e.g., systematically, non-systematically)	None.
Participant timeline	18	Time schedule of enrollment, interventions (including any run-ins and washouts), assessments, and visits for participants. A schematic diagram is highly recommended (see Figure)	The stepped-wedge-cluster randomized design includes 12 nursing homes. After an initial control phase of 6 months without any intervention, 4 nursing homes proceed to the intervention phase in 3 stages at 6-month intervals. An average of 15 people are enrolled per cluster and period, i.e. a total of 720 people. With regard to the relatives, the aim is to measure one relative per resident, i.e. to measure 720 people and with regard to the employees it is planned to assess 180 people.<br>The primary and secondary endpoints of the residents will be assessed at three measurement points T0/T1/T2 after 0, 12 and 24 weeks. The relatives will be assessed at the same measurement points as the resident. The employees will be assessed at every T0 of each period and at T2 of the last period.
Sample size	19	How sample size was determined, including all assumptions supporting the sample size calculation	Based on preliminary qualitative studies, we expect 15-40 new admissions to nursing homes over a six-month period, of which 15 can be included in the study for evaluation. After deducting an expected dropout rate of 30%, this leaves approximately 10 people per cluster per period or a total of 480 people. With this sample size, a two-sided Wald test has 80% power (α=5%) to detect a change in the SINGER score for everyday skills (primary outcome) due to the intervention by an average of 0.4 standard deviations (effect size = 0.4) with an intraclass correlation coefficient of 0.01, which is typical for this field. PASS 2019 Power Analysis and Sample Size Software was used for power calculation.
Recruitment	20	Strategies for achieving adequate participant enrollment to reach target sample size	The study nurse implemented in each home (employed 6 months before to 3 months after the survey phases; a total of 33 months regardless of randomisation status) acts as a central link within the facility, enabling the targeted recruitment of patients. Alternative strategies for achieving the target sample size could be increasing the number of cases in parallel facilities and, if necessary, recruiting additional facilities. The causes of any recruitment problems will be analysed at an early stage through

Section/Item	Item	Description	
			the qualitative investigations into implementation barriers and solutions will be developed.
**Methods: Assignment of interventions**			
Randomization:			
Sequence generation	21a	Who will generate the random allocation sequence and the method used	Scientific staff of the University of Potsdam
	21b	Type of randomization (simple or restricted) and details of any factors for stratification. To reduce predictability of a random sequence, other details of any planned restriction (e.g., blocking) should be provided in a separate document that is unavailable to those who enroll participants or assign interventions	During the baseline period, randomisation will determine which rural and urban nursing homes will begin the intervention at which of the three points in time. Randomisation will be carried out as stratified block randomisation using Castor EDC software (Castor EDC. (2019). Castor Electronic Data Capture. [online] Available at: https://castoredc.com.).
Allocation concealment mechanism	22	Mechanism used to implement the random allocation sequence (e.g., central computer/telephone; sequentially numbered, opaque, sealed containers), describing any steps to conceal the sequence until interventions are assigned	The randomization allocation will be communicated to each center enrolling in the intervention 3-4 months prior to its initiation. The randomization process will be communicated to the staff responsible one month prior to the intervention, through the SGB-Reha specific training. Randomization allocation will otherwise only be known to scientific staff at the University of Potsdam.
Implementation	23	Whether the personnel who will enroll and those who will assign participants to the interventions will have access to the random allocation sequence	Not applicable
Blinding	24a	Who will be blinded after assignment to interventions (e.g., participants, care providers, outcome assessors, data analysts)	Raters (assessors) responsible for the data collection, which are not members of the nursing home staff

Section/Item	Item	Description	
	24b	If blinded, how blinding will be achieved and description of the similarity of interventions	The raters are blinded in that the aim of the study is concealed from them (specifically, the psychometric validation of the parameters collected is stated as the aim of the study) and the study nurses on site conceal the current intervention status of the respective nursing home. All employees, patients and relatives involved in the trial will be instructed not to reveal the interventional nature of the trial to the raters.
	24c	If blinded, circumstances under which unblinding is permissible, and procedure for revealing a participant's allocated intervention during the trial	Not applicable
**Methods: Data collection, management, and analysis**			
Data collection methods	25a	Plans for assessment and collection of trial data, including any related processes to promote data quality (e.g., duplicate measurements, training of assessors) and a description of trial instruments (e.g., questionnaires, laboratory tests) along with their reliability and validity, if known. Reference to where data collection forms can be accessed, if not in the protocol	Assessors will be trained in the assessment of all relevant variables. (Check for reliability/plausibility from University of Potsdam)<br><br>Next, we describe the study instruments for the nursing *home residents*.<br>**For T0 - Baseline:**<br>- Sociodemographic information: age, gender/sex, marital status, children, immigration background, native language, education, occupation, living arrangements, nicotine consumption, alcohol consumption, falls, level of care, dominant hand<br>- For the anamnesis:<br>○ The expanded version of the Charlson's comorbidity index (CCI) (Charlson, Pompei et al., 1987) for assessing comorbidities<br>○ Hospital stays in the last 12 months<br>○ Neurological evaluation<br>- The Scores of Independence for Neurologic and Geriatric Rehabilitation (SINGER) to assess ADL skills<br>- The Tilburg Frailty Indicator (TFI) for frailty<br>- The Montreal Cognitive Assessment (MoCA) for Cognition<br>- The 12-Item Short Form Survey (SF-12) for health-related quality of life

Section/Item	Item	Description	
			- The 20-Cents-Test to assess fine-motor skills<br>- The WHOQOL-OLD for general quality of life<br>- The hand dynamometer MAP 80K1 from KERN & Sohn GmbH for measuring the grip strength<br>- The Mini Nutritional Assessment (MNA) for assessing the nutritional state<br>- The intake of medication to evaluate the intake of psychotropic drugs and polypharmacy.<br><br>**For T1 - 3 months after baseline:**<br>- Number of hospital stays in the last 3 months<br>- The Scores of Independence for Neurologic and Geriatric Rehabilitation (SINGER) to assess ADL skills<br>- The Tilburg Frailty Indicator (TFI) for frailty<br>- The Montreal Cognitive Assessment (MoCA) for Cognition<br>- The 12-Item Short Form Survey (SF-12) for health-related quality of life<br>- The 20-Cents-Test to assess fine-motor skills<br>- The WHOQOL-OLD for general quality of life<br>- The hand dynamometer MAP 80K1 from KERN & Sohn GmbH for measuring the grip strength<br>- The Mini Nutritional Assessment (MNA) for assessing the nutritional state<br>- The intake of medication to evaluate the intake of psychotropic drugs and polypharmacy.<br><br>**For T2 - 6 months after baseline:**<br>- Number of hospital stays in the last 3 months<br>- The Scores of Independence for Neurologic and Geriatric Rehabilitation (SINGER) to assess ADL skills<br>- The Tilburg Frailty Indicator (TFI) for frailty<br>- The Montreal Cognitive Assessment (MoCA) for Cognition<br>- The 12-Item Short Form Survey (SF-12) for health-related quality of life<br>- The 20-Cents-Test to assess fine-motor skills

Section/Item	Item	Description	
			- The WHOQOL-OLD for general quality of life<br>- The hand dynamometer MAP 80K1 from KERN & Sohn GmbH for measuring the grip strength<br>- The Mini Nutritional Assessment (MNA) for assessing the nutritional state<br>- The intake of medication to evaluate the intake of psychotropic drugs and polypharmacy.<br><br>Study instruments for the *relatives*:<br>**For T0 at baseline, T1 3 months after baseline and T2 6 months after baseline:**<br>- WHOQOL-BREF to assess the general quality of life.<br><br>Study instruments for the *employees*:<br>**For T0 at baseline and every 6 months at the start of each new period:**<br>- The Copenhagen Psychosocial Questionnaire (COPSOQ) to measure job satisfaction.<br><br>Reliability:<br>The SINGER is based on the International Classification of Functioning, Disability and Health (ICF) and assesses 20 aspects of independence in daily activities of living. The development study reported excellent internal consistency (Cronbach's α = 0.97) and interrater reliability (ICC = 0.996) and demonstrated strong convergent validity with other commonly used rating instruments, including the Barthel Index (BI) (r = 0.907 with the physical activities factor of the SINGER) and the Functional Independence Measure (FIM) (r = 0.980) (6). Compared with the BI and FIM, the SINGER showed considerably reduced ceiling effects in an outpatient neurorehabilitation setting, indicating good discriminative capacity (7).
	25b	Plans to promote participant retention and complete follow-up, including list of any outcome data to be collected for participants who discontinue or deviate from intervention protocols	None.

Section/Item	Item	Description	
Data management	26	Plans for data entry, coding, security, and storage, including any related processes to promote data quality (e.g., double data entry; range checks for data values). Reference to where details of data management procedures can be accessed, if not in the protocol	**Data Entry and Coding**<br>Data entry and coding are conducted utilizing secuTrial®, a certified data capture system compliant with Good Clinical Practice (GCP) standards and FDA 21 CFR Part 11. The system automates the generation of pseudonyms (Study-ID) for new study participants upon enrollment, ensuring consistency and minimizing errors. Secure transmission between tablets and the secuTrial® server is maintained through 128-bit SSL encryption, protecting data integrity during transfer. The Study Nurses in respective facilities maintain a specialized study identification list, containing pseudonyms, full names, birth dates, addresses, phone numbers, and enrollment dates. These lists allow for precise identification necessary for follow-up assessments. Measures to reduce errors include strict adherence to coding protocols and regular updates by Study Nurses.<br><br>**Data Security**<br>Data security is paramount, with all study data and participant information secured by multifactor authentication processes for authorized study personnel only. Access to the study server is restricted, ensuring that personnel can only view and manage data specific to their assigned roles and permissions. Transmission of sensitive information, such as insurance numbers and health data, employs encrypted channels using Cryptshare. Furthermore, separation of contact data from study data is rigorously maintained by storing them in separate, secure cabinets, reducing risk of unauthorized access or mishandling.<br><br>**Data Storage and Time Frame**<br>Data collected throughout the study are stored exclusively on servers located in Germany, in compliance with federal and regional data protection laws (BlnDSG, BbgDSG) and General Data Protection Regulation (GDPR). The data undergo a mandatory storage period of ten years following the conclusion and publication of the study results, as per the standards of Good Scientific Practice. Post-study, the secuTrial® license expires, ensuring the removal of all data from its servers subsequent to export. Two copies of the anonymized study data archive are secured,

Section/Item	Item	Description	
			with password protection, within the University of Potsdam for the stipulated duration. These data management procedures ensure the confidentiality, integrity, and availability of study data while facilitating effective analysis and reporting. For full information on data management practices, please refer to the study Data Management Plan, not included in this protocol.
Statistical methods	27a	Statistical methods used to compare groups for primary and secondary outcomes, including harms	**Main Analysis Method for Statistical Comparison:**<br>Statistical analysis will utilize repeated-measures linear mixed-effects multilevel models, incorporating nursing home clusters and periods as random effects to account for intra-cluster correlation.<br>The statistical models will provide effect estimates for the intervention across primary and secondary endpoints including, basic and instrumental activities of daily living, frailty, cognitive function, hand strength, fine motor skills, health-related quality of life, nutritional state, job satisfaction of the employees and general quality of life of the relatives. These estimates will be reported with 95% confidence intervals to quantify the precision of effect sizes and allow for interpretation of statistical significance. Analyses will be conducted at a standard significance level of 0.05, with adjustments for multiple testing where necessary. Sensitivity analyses will include interaction terms to examine potential heterogeneity in intervention effects across different participant groups.<br>All primary and secondary analyses will adjust for age and sex, with additional covariate adjustments assessed as needed. Both adjusted and non-adjusted analyses are planned, with a primary focus on adjusted analyses for robust outcome assessment.<br><br>**Health economic analysis:**<br>Costs at the quarterly level are evaluated using repeated-measures linear mixed-effects models, with further analysis on patient-level costs and cost-effectiveness within the subset of AOK-insured participants. These analyses involve generalized linear models with Gamma distribution to assess intervention impact on expenditures, further expanded in sensitivity analyses to validate decisional factors behind the cost-effective measures identified.

Section/Item	Item	Description	
			**Qualitative and Process Evaluations:**<br>The qualitative process evaluation identifies barriers and facilitators for implementation on the contextual, organizational, and individual level. Data is gathered in four one-week observation periods per facility prior to and during each intervention phase. During these, semi-structured interviews and the Organizational Readiness for Implementing Change (ORIC) questionnaire are conducted with staff members, and observations of work routines and intervention measures made. Barriers and facilitators are analyzed using the Consolidated Framework for Implementation Research (CFIR).<br><br>**Qualitative Outcome Evaluation:**<br>For the qualitative outcome evaluation, three one-week observation periods per facility are conducted at the end of each intervention phase. The interventions' subjective impact on study participants will be captured and analyzed through semi-structured interviews with participants, relatives and staff, and participant observations in the facilities. Qualitative data is analyzed deductively and inductively in MAXQDA to evaluate anticipated as well as unexpected intended and unintended outcomes and revise the intervention's theory of change model (8).<br><br>The results of the process and qualitative outcome analysis will be analyzed for congruence with quantitative results. This will facilitate a holistic evaluation of intervention success and long-term applicability.
	27b	Definition of who will be included in each analysis (e.g., all randomized participants), and in which group	**Participants Included in Primary and Other Analyses**<br>The analysis will encompass all participants who were randomized, ensuring adherence to the Intention-to-Treat (ITT) principle. This approach includes every participant allocated to the treatment or control group, which ensures that the broad picture of intervention effectiveness is assessed without excluding those for reasons related to post-randomization issues. The comprehensive inclusion ensures that the study findings are reflective of the diverse participant experiences within the

Section/Item	Item	Description	
			randomized framework.<br><br>**Trial Group Analysis:**<br>Participants will be analyzed within the trial groups to which they were initially randomized. This "as-randomized" analytical approach minimizes biases that could arise from post-assignment variations, maintaining the research's statistical power and validity. By analyzing according to initial trial group assignment, the study mitigates confounding variables associated with intervention compliance and external influences.<br><br>**Exclusions Due to Missing Data or Other Reasons:**<br>There will be no exclusions based explicitly on the presence of missing data, as all missing data are expected to be accounted for through established data handling methods. The analysis aims to be as inclusive as possible, considering data collection integrity. However, exclusion from analyses would only be considered in exceptional cases where data is severely compromised or completely absent, impacting the evaluation of specific study parameters or adherence monitoring.
	27c	How missing data will be handled in the analysis	The strategy for handling missing data in this study is based on the assumption that the data are Missing at Random (MAR). Missing data will be addressed using Multiple Imputation by Chained Equations (MICE). This method is chosen for its capability to handle different types of variables (e.g., continuous, binary) within the dataset, allowing for the generation of multiple datasets with filled-in missing values. It maintains power and reduces bias by considering the uncertainty associated with imputing missing values, offering a robust alternative to complete case analyses.<br>For each analysis, methods to manage potential discrepancies due to missing data will remain consistent with the outlined imputation strategy. Sensitivity analyses will be conducted to assess the impact of missing data assumptions and handling approaches on study outcomes, ensuring that conclusions are reliable and accurately reflect intervention effects. Through these methods, the study aims to uphold the integrity of data analysis despite potential challenges posed by incomplete datasets.

Section/Item	Item	Description	
	27d	Methods for any additional analyses (e.g., subgroup and sensitivity analyses)	Additional analyses, including subgroup and sensitivity analyses, are not pre-specified at this stage. Should the need arise, any such analyses will be defined and documented prior to unblinding and reported transparently alongside the primary results.
**Methods: Monitoring**			
Data monitoring committee	28a	Composition of data monitoring committee (DMC); summary of its role and reporting structure; statement of whether it is independent from the sponsor and funder; conflicts of interest and reference to where further details about its charter can be found, if not in the protocol. Alternatively, an explanation of why a DMC is not needed	The external Safety-Board consisted of two national experts in Gerontopsychiatry from the “Deutsche Akademie für Gerontopsychiatrie und -psychotherapie e.V.” (DAGPP):<br>- *Univ.-Prof. Dr. med. Andreas Fellgiebel*<br>- *Dr. med. Jochen Tenter*<br><br>The members of this board are independent of the sponsor and trial investigators and have no competing interest.<br><br>The main task of the Safety Board is the regular review of patient safety within the framework of the intervention. For this purpose, the members of the Safety Board receive statistical evaluations at specified intervals regarding potentially occurring adverse events reported by the participating nursing homes and check if these occur significantly more frequently in the intervention group provided by the Institute of Biometry and Registry Research (MHB) under the leadership of Prof. Dr. Michael Hauptmann.
	28b	Explanation of any interim analyses and stopping guidelines, including who will have access to these interim results and make the final decision to terminate the trial	The final decision to terminate the trial corresponds to the Safety-Board in conjunction with the consortium leader and project management.
Trial monitoring	29	Frequency and procedures for monitoring trial conduct. If there is no monitoring, give explanation	The feasibility of the project will be checked by the Safety-Board every six months.

Section/Item	Item	Description	
**Ethics**			
Research ethics approval	30	Plans for seeking research ethics committee/institutional review board approval	The study was approved by the Ethics Committee of the Brandenburg Medical School Theodor Fontane (Medizinische Hochschule Brandenburg Theodor Fontane, MHB), Neuruppin, Germany (reference number: E-02-20220930; approval date: 19 December 2022).<br><br>Any substantial protocol modifications that may affect participant safety, study procedures, eligibility criteria, outcomes, or data management will be submitted to the responsible ethics committee for review and approval prior to implementation.<br><br>The study is conducted in accordance with the Declaration of Helsinki, Good Clinical Practice (GCP) principles, and applicable German data protection regulations, including the General Data Protection Regulation (GDPR).
Protocol amendments	31	Plans for communicating important protocol modifications to relevant parties	Any important protocol modifications that may affect the study design, participant safety, eligibility criteria, intervention procedures, outcomes, statistical analyses, or data management will be documented and communicated to the relevant parties in a timely manner.<br><br>Substantial amendments will be submitted to the responsible ethics committee for approval prior to implementation. Relevant modifications will additionally be communicated to the trial sponsor, participating nursing homes, investigators, and study personnel as appropriate.<br><br>Where applicable, changes affecting trial registration information will be updated in the ISRCTN registry (ISRCTN11686133). Important protocol amendments will also be reported in future scientific publications arising from the study.
Consent or assent	32a	Who will obtain informed consent or assent from potential trial participants or authorized proxies, and how	Written informed consent will be obtained from all study participants prior to enrollment in the study.<br><br>For nursing home residents, informed consent will be obtained by trained study personnel or study nurses at the participating facilities after provision of oral and

Section/Item	Item	Description	
			written study information. Capacity to consent will be assessed prior to enrollment. Residents who are unable to provide informed consent due to severe cognitive impairment or dementia-related conditions will not be included in the study.<br>Relatives and nursing home staff participating in the study will likewise receive written and oral information regarding study procedures, data protection, voluntariness of participation, and the right to withdraw at any time without disadvantage. Written informed consent will be obtained prior to participation.<br>Participants will be given sufficient time to consider participation and to ask questions before signing the consent form. Signed consent forms will be stored securely and separately from study data in accordance with data protection regulations.
	32b	Additional consent provisions for collection and use of participant data and biological specimens in ancillary studies, if applicable	No biological specimens are collected as part of this study.<br>Additional consent is obtained from participants insured with AOK Rheinland/Hamburg for the use and linkage of routine health insurance data for health-economic analyses.<br>Participants are informed about the scope, purpose, storage, and processing of study data within the participant information materials and consent forms. Consent for participation is voluntary and may be withdrawn at any time without disadvantage.<br>No consent for future use of identifiable participant data in ancillary studies or for public data sharing is requested.
Confidentiality	33	How personal information about potential and enrolled participants will be collected, shared, and maintained in order to protect confidentiality before, during, and after the trial	Personal information of potential and enrolled participants will be collected, processed, and stored in accordance with the General Data Protection Regulation (GDPR), applicable German data protection laws, and institutional data protection policies.<br>All study participants are assigned a unique pseudonymized study identification number upon enrollment using the certified electronic data capture system

Section/Item	Item	Description	
			secuTrial®. Identifying information (e.g., names, addresses, dates of birth, and contact details) is stored separately from study data in secured identification lists maintained at the participating facilities by authorized study personnel only.<br>Electronic study data are entered and managed within secuTrial®, which complies with Good Clinical Practice (GCP) standards and FDA 21 CFR Part 11 requirements. Data transmission is encrypted using secure SSL protocols, and access to study data is restricted through password-protected, role-based authorization procedures and multifactor authentication.<br>Sensitive information and routine health insurance data are transferred exclusively through encrypted communication channels. Study data are stored on secure servers located in Germany in accordance with federal and regional data protection regulations.<br>Only authorized study personnel have access to identifiable participant information. Data used for statistical analyses are pseudonymized. Results will be reported in aggregated form only, ensuring that individual participants cannot be identified.<br>Study data will be retained for ten years following study completion and publication in accordance with principles of Good Scientific Practice, after which data will be archived or deleted according to institutional and legal requirements.
Ancillary and post-trial care	34	Provisions, if any, for ancillary and post-trial care, and for compensation to those who suffer harm from trial participation	The intervention evaluated in this study is non-pharmacological and based on established rehabilitative and psychosocial care approaches routinely used in geriatric and nursing home care. No major risks or harms directly attributable to study participation are anticipated.<br>Participants continue to receive standard medical and nursing care throughout the study irrespective of participation status. The intervention is implemented within existing care structures of the participating nursing homes and does not replace routine healthcare provision.<br>An independent external Safety Board regularly reviews participant safety and monitors adverse events during the trial. Any adverse events occurring during the

Section/Item	Item	Description	
			study period will be documented and evaluated.<br>No specific financial compensation is provided for study participation. In the unlikely event that participants suffer harm directly related to study procedures, appropriate medical care will be ensured in accordance with applicable legal and institutional regulations.

References

(1) Destatis Statistisches Bundesamt (2024) 5,7 Millionen Pflegebedürftige zum Jahresende 2023. Pressemitteilung Nr. 478 vom 18. Dezember 2024 [Accessed March 25, 2026]

(2) Schwinger, A, Kuhlmey, A, Greß, S, Klauber, J, & Jacobs, K (2023). Pflege-Report 2023: Versorgungsqualität von Langzeitgepflegten (p. 289). Springer Nature.

(3) Onder, G., Liperoti, R., Fialova, D., Topinkova, E., Tosato, M., Danese, P., ... & Landi, F. (2012). Polypharmacy in nursing home in Europe: results from the SHELTER study. Journals of Gerontology Series A: Biomedical Sciences and Medical Sciences, 67(6), 698-704.

(4) Xu, M, Yan, G. (2025) Length of stay in residential aged care: Patterns and determinants from a population-based cohort study. Insurance: Mathematics and Economics, 122:214-229 https://doi.org/10.1016/j.insmatheco.2025.03.006.

(5) Kolip, P, Güse, HG. Kurzzeitpflege nach Krankenhausaufenthalt: Einflussfaktoren auf die Entlassung in die eigene Häuslichkeit. Z Gerontol Geriat (2004), 37:231–239. https://doi.org/10.1007/s00391-004-0188-3

(6) Crocker T, Forster A, Young J, Brown L, Ozer S, Smith J, Green J, Hardy J, Burns E, Glidewell E, Greenwood DC. Physical rehabilitation for older people in long-term care. Cochrane Database Syst Rev. 2013 Feb 28;2013(2):CD004294. doi: 10.1002/14651858.CD004294.pub3. PMID: 23450551; PMCID: PMC11930398.

(7) Kieslich, B. (2011). Pflegeheim-Einbahnstraße?. [dissertation]. [Nürnberg, Germany] Friedrich-Alexander-Universität Erlangen-Nürnberg

(8) Desch A, Förstner B, Artmann J, Häusler A, Hauptmann M, Altin S, Rapp M, Holmberg C. A theory of change of an innovation for therapeutic care and meaningful living in a German nursing home. BMC Geriatr. 2022 Nov 11;22(1):849. doi: 10.1186/s12877-022-03462-0. PMID: 36368919; PMCID: PMC9651899.

(9) Romero, B, & Wenz, M (2003). Wirksame Behandlungsprogramme für Demenzkranke und betreuende Angehörige. Neurologie und Rehabilitation, 9(1), 23-27.

(10) Oswald, W, Ackermann, A & Gunzelmann, T (2006). Effekte eines multimodalen Aktivierungsprogrammes (SimA-P) für Bewohner von Einrichtungen der stationären Altenhilfe. Zeitschrift für Gerontopsychologie & -psychiatrie. 19. 10.1024/1011-6877.19.2.89.
